# Mannosylated glycans impair normal T-cell development by reprogramming commitment and repertoire diversity

**DOI:** 10.1038/s41423-023-01052-7

**Published:** 2023-06-21

**Authors:** Manuel M. Vicente, Inês Alves, Ângela Fernandes, Ana M. Dias, Beatriz Santos-Pereira, Elena Pérez-Anton, Sofia Santos, Tao Yang, Alexandra Correia, Anja Münster-Kühnel, Afonso R. M. Almeida, Sarina Ravens, Gabriel A. Rabinovich, Manuel Vilanova, Ana E. Sousa, Salomé S. Pinho

**Affiliations:** 1 i3S – Institute for Research and Innovation in Health, University of Porto, Porto, Portugal; 2 Institute of Biomedical Sciences Abel Salazar (ICBAS), University of Porto, Porto, Portugal; 3 Graduate Program in Areas of Basic and Applied Biology (GABBA), ICBAS, University of Porto, Porto, Portugal; 4 Faculty of Medicine, University of Porto, Porto, Portugal; 5 Nephrology Department, Centro Hospitalar e Universitário do Porto, Porto, Portugal; 6 Institute of Immunology, Hannover Medical School, Hannover, Germany; 7 Institute of Clinical Biochemistry, Hannover Medical School, Hannover, Germany; 8 Instituto de Medicina Molecular João Lobo Antunes, Faculdade de Medicina, Universidade de Lisboa, Lisboa, Portugal; 9 Laboratorio de Inmunopatología, Instituto de Biología y Medicina Experimental (IBYME), Consejo Nacional de Investigaciones Científicas y Técnicas (CONICET), Ciudad de Buenos Aires, Argentina; 10 Laboratorio de Inmuno-oncología Translacional, Instituto de Biología y Medicina Experimental (IBYME), Consejo Nacional de Investigaciones Científicas y Técnicas (CONICET), Ciudad de Buenos Aires, Argentina; 11 Facultad de Ciencias Exactas y Naturales (FCEyN), Universidad de Buenos Aires, Ciudad de Buenos Aires, Argentina

**Keywords:** *N*-glycosylation, T-cell development, Thymocytes, Glycocalyx, Inflammation, Adaptive immunity, Lymphopoiesis, Autoimmunity

## Abstract

T-cell development ensures the formation of diverse repertoires of T-cell receptors (TCRs) that recognize a variety of antigens. Glycosylation is a major posttranslational modification present in virtually all cells, including T-lymphocytes, that regulates activity/functions. Although these structures are known to be involved in TCR-selection in DP thymocytes, it is unclear how glycans regulate other thymic development processes and how they influence susceptibility to disease. Here, we discovered stage-specific glycome compositions during T-cell development in human and murine thymocytes, as well as dynamic alterations. After restricting the *N*-glycosylation profile of thymocytes to high-mannose structures, using specific glycoengineered mice (*Rag1*^Cre^*Mgat1*^fl/fl^), we showed remarkable defects in key developmental checkpoints, including ß-selection, regulatory T-cell generation and γδT-cell development, associated with increased susceptibility to colon and kidney inflammation and infection. We further demonstrated that a single *N*-glycan antenna (modeled in *Rag1*^Cre^*Mgat2*^fl/fl^ mice) is the *sine-qua-non* condition to ensure normal development. In conclusion, we revealed that mannosylated thymocytes lead to a dysregulation in T-cell development that is associated with inflammation susceptibility.

## Introduction

Thymic T-cell development is a tightly regulated process that ensures the formation of a T-cell pool with a diverse repertoire of T-cell receptors (TCRs), essential in adaptive immunity [1, 2]. Thymocytes pass through a series of developmental stages, mainly defined by variations in coreceptor expression. In the CD4^-^CD8^-^ double-negative-3 (DN3) stage, cells commit to the αß or γδ lineages. Then, massive proliferation of DN4 cells leads to the generation of CD4^+^CD8^+^ double-positive (DP) thymocytes, where a successful rearrangement of the *Tcra* locus generates a mature and cell-unique TCR. An intermediate population is found on the DN4-to-DP transition, the CD8^+^CD3^−^ (mouse) or CD4^+^CD3^−^ (human) immature-single-positive (ISP) cells. Development of DP into mature CD4^+^ or CD8^+^ single-positive (SP) thymocytes is highly regulated by two steps: “positive-” and “negative-selection”, where non- or self-reactive T-cells are eliminated. Moreover, cells with TCR affinities close to the threshold for negative selection develop into natural regulatory T-cells (nTregs) [3].

Glycans are present on essentially all cellular surfaces and are important regulators of the immune system. In fact, T-cells contain a dense coat of glycans (glycocalyx) that tightly regulate activity and function [4–6]. Glycosylation is the enzymatic pathway responsible for the attachment of glycans to proteins/lipids by a portfolio of glycosyltransferase and glycosidase enzymes [7]. Protein glycosylation has been shown to be essential in T-cell activation and differentiation [8–11]. However, its precise impact on T-cell development and disease susceptibility remains poorly understood. Previous evidence has demonstrated essential contributions of α2,3-linked sialic acid to T-cell development [12]. Furthermore, seminal work showed *N*-glycan branching to be a master regulator of DP-selection, tuning the thresholds of TCR signaling [13]. However, the role of *N*-glycans in other thymic developmental processes, such as ß-selection, γδT-cell development, natural Treg generation, and the peripheral consequences of a central deficiency of these structures (disease susceptibility), remains undefined. In fact, complex-branched *N*-glycans have been described as chief regulators of T-cell activity and function [4]. Specifically, we and others have shown that the deficiency of β1,6-GlcNAc-branched complex *N*-glycans promotes TCR clustering and signaling, resulting in lower activation thresholds associated with increased susceptibility to multiple sclerosis [14] and inflammatory bowel disease [10, 11, 15].

Overall, this evidence identifies *N*-glycans as key regulators of peripheral T-cell activity and function, but knowledge of their role in central tolerance and T-cell development associated with disease is still unclear. In particular, thymocyte glycocalyx composition and its influence on disease susceptibility remain largely unexplored. Here, we showed that human and murine thymocyte subsets display unique and distinct glycosylation signatures that define developmental stages. We further demonstrated that high-mannose restricted *N*-glycomes in thymocytes globally impair their normal development, predominantly in ß-selection, Treg cell generation and γδT-cell lineage choice and selection, which was associated with spontaneous autoimmune phenotypes. In addition, we showed that a single *N*-acetylglucosamine (GlcNAc) antenna is the *sine-qua-non* condition needed to rescue homeostasis and T-cell developmental dynamics and peripheral phenotypes due to a glycosylation pathway compensatory effect.

## Materials and methods

### Single-cell RNA sequencing data processing

Two publicly available single-cell RNA sequencing datasets of human and murine thymocytes [16] were downloaded from the Human Developmental Cell Atlas web portal under accession number E-MTAB-8581. Quality control was performed using *scanpy* version 1.7.2, which included filtering out cells with fewer than 200 genes detected and genes expressed in fewer than 3 cells. Clustering analysis was already performed and visualized using uniform manifold approximation and projection (UMAP) dimensionality reduction. For the murine thymocyte dataset, samples from embryonic stages were removed for final analysis. Data visualization of glycogene expression was performed using *scanpy*’s built-in plotting functions, including UMAP and dot plots.

### Animals

C57BL/6 wild-type (WT) mice were acquired from the Jackson laboratory. Mice containing floxed *Mgat1* (JAX:006891) and *Mgat2* (JAX:006892) were kindly provided by Dr. Michael Demetriou (UC Irvine, USA). *Rag1*^Cre^ transgenic mice, with a *Cre* recombinase gene introduced under the promoter of the *Rag1* gene [17], were kindly provided by Dr. Marc Veldhoen (IMM, Portugal). Mice (males) with two floxed alleles for *Mgat1* or *Mgat2* were crossed with *Rag1*^Cre^ mice (females), with only one *Cre* allele, to generate *Rag1*^Cre^ with heterozygous floxed (both for *Mgat1* or *Mgat2*) progeny. These mice (females) were then crossed with homozygous floxed *Mgat1* or *Mgat2* mice (males) to generate *Rag1*^Cre^*Mgat1*^f/f^ (*Mgat1*^*Δ/Δ*^) and *Rag1*^Cre^*Mgat2*^*f/f*^ (*Mgat2*^*Δ/Δ*^) progeny. The littermates containing only the two floxed alleles (*Mgat1*^*f/f*^ and *Mgat2*^*f/f*^) were used as controls. Mice were housed at the animal facility of the Institute for Research and Innovation in Health of the University of Porto. All mouse procedures were approved by the i3S ethics committee for animal experimentation under Portuguese regulation.

### Human thymocyte collection

Thymic specimens (173–578 days old) were obtained during routine thymectomy performed during pediatric corrective cardiac surgery at Hospital de Santa Cruz (HSC), Carnaxide, Portugal, after the parent signed a written informed consent form, using thymic tissue that would otherwise be discarded. The study was approved by the Ethics Boards of the Faculty of Medicine of the University of Lisbon and of HSC.

Total thymocytes were recovered through tissue dispersion and separation on a Ficoll-Paque PLUS (GE Healthcare) density gradient. Cells were then frozen in a drop-by-drop freezing medium (86% FCS, 14% DMSO), placed for 48 h at −80 °C in an isopropanol container and stored in liquid nitrogen until use, and the cells were thawed immediately before use.

### Human PBMC collection

Human peripheral blood mononuclear cells (PBMCs) from inflammatory bowel disease patients (*n* = 3) and healthy donors (*n* = 3) were isolated by gradient centrifugation using 1 volume of Lymphoprep^TM^ (Stemcell Technologies) for 2 volumes of blood, for 30 min at 900 × *g* with the brake off. PBMCs (interphase) were collected and frozen in FBS with 10% DMSO until further analysis.

### Mouse organ isolation and processing

After CO_2_-mediated euthanasia, thymi and spleens were collected from age- and sex-matched mice and rinsed in PBS containing 2% FBS. Organs were macerated in a 70 μm nylon mesh to generate single-cell suspensions. For erythrocyte lysis, cells were incubated for 3 min at room temperature with 1× ACK (150 mM NH_4_Cl; 10 mM KHCO3; and 0.1 nM Na_2_EDTA) and washed in PBS with 2% FBS. Cells were counted and stored in PBS with 2% FBS on ice until further immediate use. Serum was collected after centrifugation of whole blood. Colons and kidneys were flushed with PBS, opened longitudinally, cut into small pieces and digested with 0.9 mg/mL collagenase IV (Sigma) in RPMI (RPMI-1640 GlutaMAX; Gibco) supplemented with 10% fetal bovine serum (FBS; Gibco), 100 U/mL penicillin/streptomycin (Pen/Strep; Gibco), 1 mM CaCl2 and 1 mM MgCl2 for 45 min with agitation at 37 °C. Cell suspensions were then filtered in 70-μm cell strainers and washed with RPMI. Afterward, the cells were subjected to Lymphoprep (Alere Technologies) gradient density centrifugation at 900 × *g*, without acceleration or braking, for 30 min at RT. Immune cells were collected in the interphase. After washing once with PBS and centrifuging at 300 × *g* for 10 min at 4 °C, the cells were resuspended in PBS, placed in a 96-well plate, and subjected to flow cytometry staining. For cytokine-producing cell analysis, cells were incubated in a humidified atmosphere with 5% CO_2_ at 37 °C for 5 h under stimulation with 10 ng/ml phorbol myristate acetate (PMA; Sigma) and 1 μg/ml ionomycin (Merck) in the presence of 10 μg/ml brefeldin A (Sigma). A portion of colon and kidney tissue was placed in 300–500 μL of RPMI for 18 h in 48-well plates. The weight of tissue pieces was determined, and supernatants were stored at −80 °C for later analysis by ELISA.

### Flow cytometry

Cell suspensions were stained with lectins and monoclonal antibodies from eBioscience and BioLegend, as described [11]. Briefly, for viability detection, cells were resuspended in PBS and incubated with Fixable Viability Dye APC-Cy7 for 30 min on ice in the dark. For lectin staining, performed in murine and human thymocytes, 1 × 10^6^ cells were isolated and incubated with conjugated lectins for 30 min on ice prior to antibody staining. Antibodies were titrated, optimal concentrations were set, and cells were incubated for 30 min on ice in the dark. For intracellular staining, cells were fixed and permeabilized with the FoxP3 Fix/Perm buffer set (eBioscience). For TCRvß screening, cells were stained for specified markers, washed, and incubated for 30 min on ice in the dark with supplied antibody suspensions. Cells were analyzed in a FACSCanto II (BD Bioscience), and the data were analyzed with FlowJo v10 software.

### TCR amplicon generation and NGS

RNA was extracted from 20,000 FACS-sorted CD4^+^ and CD8^+^ SP thymocytes of the *Mgat1*^*f/f*^ and *Mgat1*^*Δ/Δ*^ groups (*N* = 4 per genotype) using an RNeasy Micro Kit (Qiagen). Subsequently, cDNA was synthesized using a SMARTer RACE cDNA Amplification Kit (Clontech), followed by amplification of the VDJ (CDR3) region using a 5′RACE PCR kit (Clontech) with a specific primer for the murine TCRβ locus (Trb). Primer sequences for 5’RACE PCR are as follows: (forward) RACE-long universal primer, CTAATACGACTCACTATAGGGCAAGCAGTGGTATCAACGCAGAGT; (forward) RACE-short universal primer, [Illumina Overhang]-CTAATACGACTCACTATAGGGC; and (reverse) TRB (mouse)-primer, [Illumina Overhang]-TGGCTCAAACAAGGAGACCT. The Illumina overhang adaptor sequences were as follows: forward, GTCTCGTGGGCTCGGAGATGTGTATAAGAGACAG; and reverse, TCGTCGGCAGCGTCAGATGTGTATAAGAGACAG. The resulting amplicons were subjected to Illumina MiSeq paired-end sequencing with 500 cycles.

### Data analysis

Raw fastq read files were annotated with MiXCR software based on the ImMunoGeneTics (IMGT) database. The annotated files were then processed and analyzed with the Immunarch package using R version 4.0.3. The function “repDiversity” was used to calculate the inverse Simpson index, which quantifies the average proportional abundance of types in the dataset. The function “repOverlap” was used to calculate the Morisita–Horn similarity index, which quantifies the overlap between two samples. Treemaps were generated using the R package ggplot2. Statistical analysis was performed using R, and the significance was calculated using the two-way Student’s *t* test. The mean values are shown ± standard error of the mean (SEM). *P* values ≤ 0.05 were considered statistically significant.

### ELISA and fecal calprotectin concentration evaluation

Serum and supernatants from tissue explants were collected, and the concentrations of IFNg and IL-17A (mouse ELISA Ready-SET-Go! Kits, eBioscience) were measured by ELISA according to the manufacturer’s protocol using 3,3′,5,5′-tetramethylbenzidine (TMB, eBioscience) as the substrate and a stop solution of 2 N H2SO4. The absorbance was detected using a microplate reader (Biotek Instruments) at 450 nm and 570 nm, the concentration was determined using a standard curve, and the results were posteriorly normalized to the tumor explant dry weight (g). For fecal calprotectin concentration determination, feces were collected and weighed, and the protein was extracted using an extraction buffer (0.1 M Tris, 0.15 M NaCl, 1.0 M urea, 10 mM CaCl2, 0.1 M citric acid monohydrate, and 5 g/l BSA – pH 8.0). Then, the levels of calprotectin (mouse S100A8/A9 Heterodimer DuoSet ELISA kit, R&D systems) were measured by ELISA according to the manufacturer’s protocol.

### Tissue histology

Colon and kidney samples were immediately fixed in 10% neutral buffered formalin FFPE sample blocks, sectioned at 3 µm using a microtome (Microm HM335E microtome) and placed on slides. These slides were used for hematoxylin and eosin (H&E) staining (colon) and periodic acid-Schiff (PAS) staining (kidney). The kidney PAS-stained sections were evaluated by a pathologist (SS) using the following protocol: a semiquantitative analysis was performed and translated into a score of 0–3. The percentage of interstitial inflammation and mesangial inflammation was scored in the following way: <5% → grade 0; 5–25% → grade 1, 25%–50% → grade 2, or >50% → grade 3. Finally, the total score is given by the sum of each criteria score/grade.

The skin and ear samples of each mouse were sectioned at 3 μm using a microtome (180 Microm HM335E), placed on slides, and then stained with hematoxylin and eosin to evaluate the detailed histological features of psoriasis. To classify the disease activity, an already described psoriasis inflammatory score was evaluated at the site of imiquimod application. The disease activity includes the following parameters: mouse body weight loss (0–5%, score 0; 5–10%, score 1; 10–15%, score 2; and above 15%, score 3), erythema (redness), induration (thickness) and desquamation (scale) on the back skin of each mouse. Each parameter was given a score of 0–3 (0, none; 1, mild; 2, moderate; and 3, severe).

### Imiquimod-induced psoriasis

Five female *Mgat1*^f/f^ and *Mgat1*^Δ/Δ^ mice between 7 and 8 weeks old were used for experimental induction of psoriasis. The back and ear of the mice were shaved and subjected to daily application of 3.125 mg of commercially available 5% imiquimod cream (Aldara, MEDA Pharmaceuticals, Solna, Sweden) under isoflurane anesthesia. The mice were euthanized, and the spleen, ear and skin (the lesion where imiquimod was applied and adjacent control tissue) were collected. The spleen was macerated in a 70 μm nylon mesh, and a splenocyte suspension was generated. For erythrocyte lysis, cells were incubated for 3 min at RT with 1× ACK and washed in PBS containing 2% FBS. The cells were counted and stored on ice until further immediate use. The ear and half of the skin samples were fixed in 4% v/v formaldehyde and then embedded in paraffin.

### *Neospora caninum* infection

*N. caninum* tachyzoites (NcT) (Nc-1, ATCC® 50843) were propagated by serial passages in VERO cell cultures, maintained in Minimal Essential Medium containing Earle’s salts (Sigma, St. Louis, MO, USA) supplemented with 10% fetal calf serum (BioWest, Nuaillé, France), L-glutamine (2 mM), penicillin (100 IU/ml) and streptomycin (100 µg/ml) (all from Sigma) in a humidified atmosphere with 5% CO2 at 37 °C. Free parasites were obtained as previously described [18]. *N. caninum* challenge infections of 8-week-old mice were performed by i.p. inoculation of 1 × 10^7^ freshly isolated NcT in 500 μL of PBS. Mouse weights and overall conditions were monitored daily until Day 7 post-infection. At euthanasia, spleens, lungs, and brains were collected for DNA isolation and parasite load quantification by qPCR.

### Quantification and statistical analysis

Statistical analyses were performed using GraphPad Prism 9 software. Further details of the statistics can be found in the figure legends. Multiple comparisons were performed using the Kruskal‒Wallis test. Significance values were computed using the Mann‒Whitney *t*-test. No statistical method was used to predetermine the sample size. All experiments were performed at least two times, with mice from independent progenies.

## Results

### The unique glycocalyx landscape in human and murine T-cell development

**Fig. 1:**
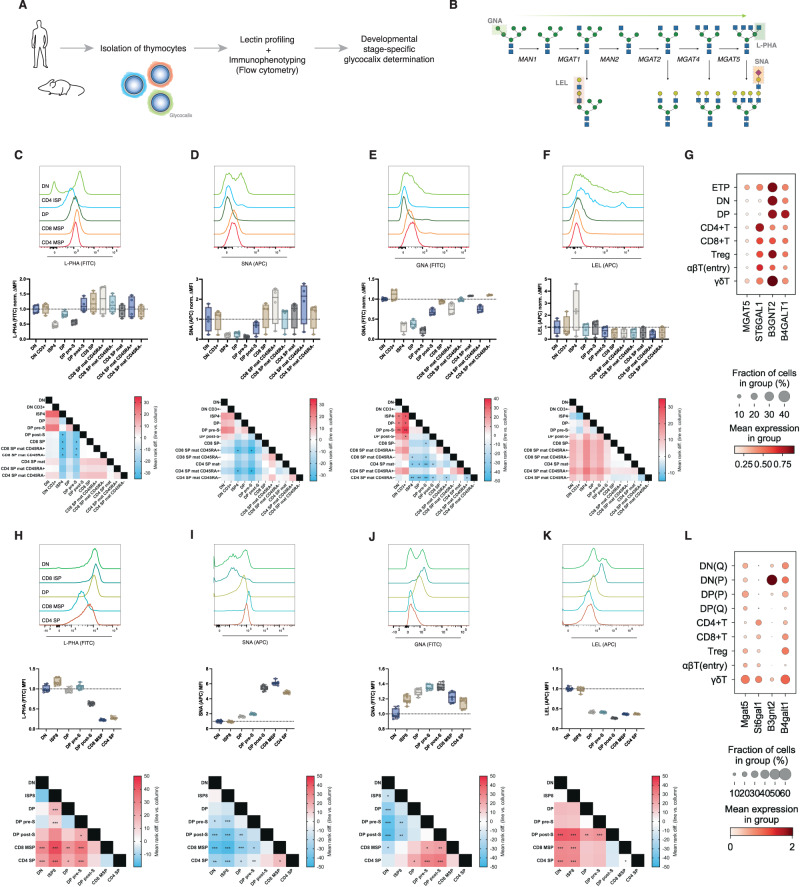
The human and murine T-cell developmental stages exhibit differential glycan profiles. **A** Scheme of the isolation of thymocytes from both human (*N* = 4) and murine (*N* = 8, 8 weeks old) thymi and subsequent workflow. **B** Major Golgi-localized steps of the *N*-glycosylation pathway, GlcNAc antennae formation, elongation and termination. Boxes indicate the specific lectin-binding glycans. **C–F** Histograms of lectin staining for major human thymocyte populations (top), median fluorescence intensity (MFI) quantifications normalized for the mean DN levels (middle) and heatmap of the mean rank differences showing the Kruskal‒Wallis test multiple comparisons between populations, presented as “population in line” vs. “population in column” (bottom), for L-PHA (**C**), SNA (**D**), GNA (**E**) and LEL (**F**). As an example for heatmap interpretation, comparison between L-PHA levels of CD8 SP and ISP4 shows a blue color, indicating a negative mean rank difference, i.e., ISP4 levels of L-PHA are lower than those of CD8 SP. **G** Analysis of *MGAT5*, *ST6GAL1*, *B3GNT2* and *B4GALT1* in a human thymocyte single-cell RNA sequencing dataset [16]. **H–K** Histograms of lectin staining for major murine thymocyte populations (top), median fluorescence intensity (MFI) quantifications normalized for the mean DN levels (middle) and heatmap of the mean rank differences showing the Kruskal‒Wallis test multiple comparisons between populations, presented as “population in line” vs. “population in column” (bottom), for L-PHA (**H**), SNA (**I**), GNA (**J**) and LEL (**K**). Kruskal‒Wallis test, *q* value * < 0.05, ** < 0.005, *** < 0.001. **L** Analysis of *Mgat5*, *St6gal1*, *B3gnt2* and *B4galt1* in a human thymocyte single-cell RNA sequencing dataset [16]

The glycocalyx composition and function of human and murine thymocytes is far from being completely deciphered. To gain insights into the composition and function of glycan structures at each T-cell developmental stage, an extensive lectin-based characterization of the human and murine thymocyte glycome was performed (Fig. 1A). A combination of different lectins recognizing specific glycan structures was used to assess the levels of relevant glycans along the *N*-glycosylation pathway (Fig. 1B). Thawed human thymocytes were used to perform glycoprofiling of the main developmental populations (Supplementary Fig. 1A). We found that thymocyte subsets display unique and stage-specific glycocalyces (Fig. 1C–F). Specifically, complex-branched *N*-glycans detected by L-PHA staining (Fig. 1C) and terminally α2,6-sialylated glycans by the SNA lectin (Fig. 1D), structures known to be involved in T-cell biology [4], displayed a clear and distinctive signature during T-cell development (Supplementary Fig. 1B). We observed differential profiles of complex-branched *N*-glycans containing ß1,6-GlcNAc antennae (L-PHA binding) across subsets, showing high representation of these *N*-glycans in DN cells, which decreased in ISP4 and finally increased in mature populations (Fig. 1C). Notably, between DP prepositive selection and both the DP postpositive selection stage and mature SP cells, we found a significant increase in L-PHA binding (Fig. 1C), which reveals differential expression dynamics of complex-branched *N*-glycans on thymocytes when compared to peripheral cells [4]. By analyzing SNA reactivity, we found α2,6-linked sialic acid enrichment in mature SP populations compared to ISP4 and DP subsets (Fig. 1D). Moreover, the levels of less complex, high-mannose *N*-glycan structures (GNA binding) were highly represented in mature SP populations, differing from ISP4 and DP populations (Fig. 1E). The presence of poly-LacNAc elongated glycans (LEL binding) was higher in the DN and ISP4 populations, reaching a peak in the latter (Fig. 1F). To further our results regarding these glycocalyx alterations, we analyzed publicly available single-cell transcriptomic datasets of human thymocytes obtained by single-cell RNA sequencing (Supplementary Fig. 1C and 1D) [16]. We focused our analysis on the expression of glycogenes that encode the glycosyltransferases related to the glycan expression profile detected by the panel of lectins used, such as *MGAT5* for L-PHA, *ST6GAL1* for SNA and *B3GNT2* and *B4GALT1* for LEL. Interestingly, we observed that the *MGAT5* glycogene exhibited a constant expression level across different developmental stages (Fig. 1G), which agrees with the L-PHA binding data. Regarding *ST6GAL1*, we observed an upregulation after the DP stage in CD4 and CD8 SP, agreeing with the SNA binding data (Fig. 1G). On the other hand, we observed a downregulation in the expression of *B3GNT2* and *B4GALT1* after the DP stage, which agrees with the LEL binding data (Fig. 1G). These results show that human thymocytes display unique and dynamic glycan signatures across development.

Then, to evaluate whether the glycan profile of murine thymocytes recapitulates that displayed in humans, we glycophenotyped biologically equivalent murine thymocyte populations (Supplementary Fig. 1E). In the mouse, the presence of complex-branched *N*-glycans (L-PHA binding) was enriched in DN, ISP8 (equivalent to human ISP4) and DP cells. As in peripheral T-cells, upon TCR signaling (positive selection), DP post-selection showed lower levels of ß1,6-GlcNAc branching than DP preselection cells (Fig. 1H). On the other hand, terminal α2,6-sialylation was found to be scarce in DN and ISP8 populations, reaching high levels in mature SP populations (Fig. 1I). Moreover, high-mannose *N*-glycans remained at (low) constant levels across all T-cell developmental stages, with differences between populations (Fig. 1J). Elongated glycans were found to have a developmental peak in ISP8 cells, being reduced from DP onward (Fig. 1K). As with human thymocytes, we analyzed the expression of the key glycogenes related to the glycan profile observed in murine thymocytes using a single-cell RNA sequencing dataset (Supplementary Fig. 1E) [16]. Interestingly, we found that *Mgat5* expression is downregulated in SP thymocytes compared to DP thymocytes, agreeing with the L-PHA binding data (Fig. 1L). Regarding *St6Gal1*, we observed an upregulation in mature SP thymocytes following the DP stages, which agrees with the SNA binding data (Fig. 1L). Finally, we found that *B3gnt2* and *B4galt1* were highly expressed in DN and DP thymocytes and were downregulated or maintained in mature SP cells (Fig. 1L). A broader panel of glycogene expression in human and murine thymocytes revealed major potential impacts of additional glycan families (Supplementary Fig. 1C and G). In fact, both human and mouse thymocytes shared major glycocalyx alterations, displaying high levels of mature complex-branched and elongated *N*-glycans in the ISP population that precedes the DP stage and increased terminal α2,6-sialylation in mature subsets. Interestingly, the relative presence of complex-branched *N*-glycans in mature SP cells (increased/maintained in humans, decreased in mice) may underlie the differences between organisms regarding thymic egress and peripheral naïve T-cell properties.

Taken together, this analysis of human and murine thymocytes suggests the presence of a developmentally regulated glycome, which supports the existence of functional effects of glycans in the regulation of T-cell development.

### High-mannose-restricted *N*-glycome significantly impairs T-cell development, which can be rescued by compensatory mono-antennary *N*-glycan structures

**Fig. 2:**
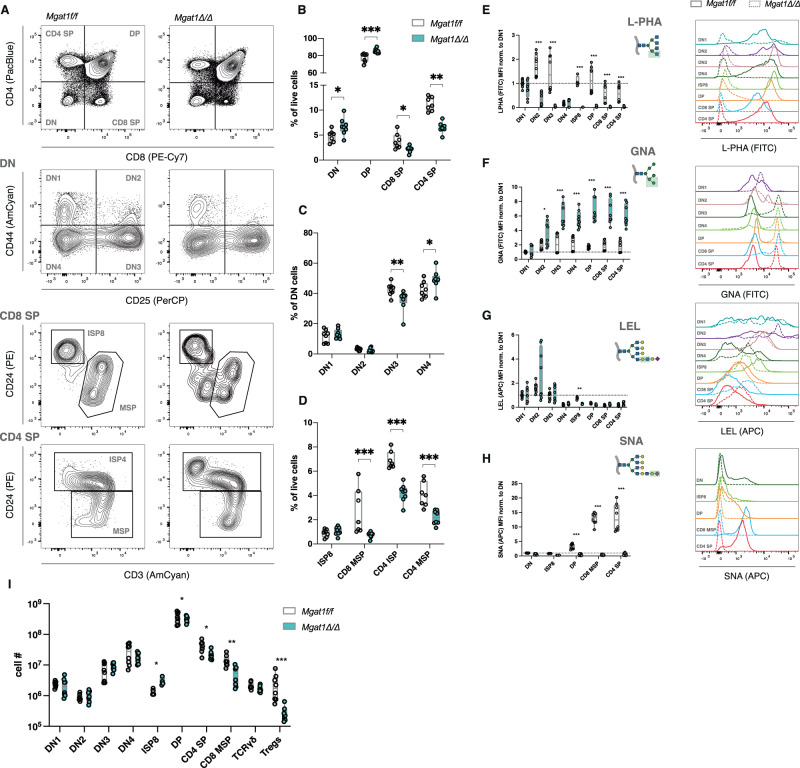
Impairment of T-cell development in high-mannose-restricted thymocytes**. A** Thymocyte population discrimination in *Mgat1*^*f/f*^ mice (*N* = 8, 6–8 weeks old) and *Mgat1*^*Δ/Δ*^ mice (*N* = 8, 6–8 weeks old). **B** Frequencies of DN, DP, CD8 SP and CD4 SP subsets within live cells. **C** Frequencies of DN1, DN2, DN3 and DN4 subsets within the total DN population. **D** CD8 SP and CD4 SP subset frequencies: ISP8, CD8 MSP, CD4 ISP and CD4 MSP within live cells. **E–H** Levels of lectin binding (MFI levels) normalized to the indicated population (left) and representative histograms of lectin binding profiles in the indicated thymocyte subsets for L-PHA (**E**), GNA (**F**), LEL (**G**) and SNA (**H**). **I** Absolute numbers of cells within the indicated populations. Each dot represents one mouse in all graphs. Mann‒Whitney *t*-test, *p* value * < 0.05, ** < 0.005 and *** < 0.001

The degree of complexity in the alterations of thymocyte *N*-glycomes during T-cell development, both in humans and mice, prompted us to investigate their functional relevance in the context of T-cell development. Previous work revealed the role of complex *N*-glycans in TCR-selection in DP thymocytes, showcasing the ability of these structures to fine-tune the thresholds of TCR signaling, thus controlling thymic DP selection [13]. This work, however, left questions to be answered, namely, the role of *N*-glycans in ß-selection, γδT-cell development, natural Treg generation, and peripheral consequences of a central deficiency of these structures (disease susceptibility). Therefore, we hypothesized that high-mannose-restricted *N*-glycomes might have a pathogenic role during T-cell development beyond DP selection. To test this hypothesis, we genetically modified the *N*-glycosylation pathway in murine models at early DN stages using a *Rag1*^Cre^ transgene strain crossed with *Mgat1*^fl/fl^ or *Mgat2*^fl/fl^ mice, thus eliminating glycogenes as early as the DN2 stage. The model with the *Rag1*^Cre^-mediated knockout of *Mgat1* (*Mgat1*^*Δ/Δ*^) has a complete truncation of complex-branched *N*-glycans, only presenting high-mannose *N*-glycans, whereas the conditional knockout of *Mgat2* (*Mgat2*^*Δ/Δ*^) exhibits a partial pathway truncation, generating hybrid-type *N*-glycans with only one GlcNAc antenna, which can be further extended. We set out to evaluate alterations in the most prevalent thymocyte populations in both glycoengineered models (Fig. 2A; Supplementary Fig. 2A). Notably, *Mgat1*^*Δ/Δ*^ mice displayed a significant increase in DN and DP thymocyte populations, which was accompanied by a significant decrease in CD8 SP and CD4 SP populations (Fig. 2B). Within the DN compartment, complete truncation of complex-branched *N*-glycans resulted in a significant drop in DN2 and DN3 subsets and an increased frequency of DN4 thymocytes (Fig. 2C), suggesting a significant role of these glycans in ß-selection. Moreover, no differences were detected in the ISP8 population, but a significant decrease in CD8 mature SP cells, CD4 immature (CD3^lo^CD24^hi^, postselection and pre-egress) and mature (CD3^hi^CD24^lo^) SP cells was observed (Fig. 2D), which may reflect increased DP negative selection, compatible with previous models of *Mgat1* conditional deletion in DP thymocytes [13]. Regarding the numbers of thymocyte populations, we detected a significant increase in ISP8 cells and a significant decrease in the CD4 and CD8 mature SP populations (Fig. 2I). Since the absence of complex-branched *N*-glycans may influence the endocytosis of cell surface glyco-receptors, we evaluated the expression levels of the CD4 and CD8 coreceptors, relevant for DP-selection/lineage commitment, and found a significant decrease in both CD4 and CD8 SP coreceptors in the respective SP populations (Supplementary Fig. 2I and J), as in previous studies [13].

We then evaluated the impact of *Mgat1* deletion on the glycocalyx composition of thymocytes. As expected with the genetically mediated interruption of the *N*-glycosylation pathway, we observed a decrease in the adhesiveness of complex-branched *N*-glycans (L-PHA binding) in all developmental stages beyond DN2 (Fig. 2E). Accordingly, we detected a remarkable increase in high-mannose *N*-glycans (GNA binding) in all developmental stages beyond DN2 (Fig. 2F). Elongated glycans (LEL binding) were unaltered in DNs, suggesting poly-LacNAc in *O*-glycans in those cells, but were severely decreased in ISP8 thymocytes (Fig. 2G). The blockade in the *N*-glycosylation pathway was also accompanied by ablation of terminal α2,6-sialic acid, as determined by SNA binding, in DP, CD4 SP and CD8 SP thymocyte glycocalyces (Fig. 2H).

These results led us to investigate whether the presence of a single GlcNAc antenna in *N*-glycans would influence normal developmental processes. Interestingly, the frequencies of thymocyte populations in *Mgat2*^*Δ/Δ*^ mice were not altered compared to those in their wild-type counterparts (Supplementary Fig. 2A–D). Thus, the presence of hybrid *N*-glycans containing the first GlcNAc antenna transferred onto the oligomannose core (creating the β-linked GlcNAc-β-1,2-Man-α-1,3-Man-β-R product) generated by GnT-I (the glycosyltransferase encoded by *Mgat1*) was sufficient and a *sine-qua-non* condition to guarantee normal thymic T-cell development. Notably, the absolute thymocyte numbers of *Mgat2*^*Δ/Δ*^ mice were unaltered for all detected populations, with the exception of ISP8 cells (Supplementary Fig. 2K). Moreover, we observed decreased expression of CD4 and CD8 coreceptor surface expression in their respective SP subsets upon *Mgat2* deficiency (Supplementary Fig. 2L and M). We then set out to define the *N*-glycome alterations upon the loss of *Mgat2*. As expected, we observed a significant decrease in complex-branched *N*-glycans (L-PHA binding) and increased levels of mannose residues (GNA binding) in all developmental stages beyond DN2 (Supplementary Fig. 2E and F). Interestingly, we found a remarkable increase in elongated poly-LacNAc structures (LEL binding) from DN3 onward (Supplementary Fig. 2G), with no significant alterations in terminal α2,6-sialylation (Supplementary Fig. 2H). Thus, the unique GlcNAc-antenna is found to be hyperelongated, similar to what was described in *Mgat2*-deficient mature T cells [19], generating a compensatory *N*-glycan structure that is sufficient to guarantee normal T-cell development.

Collectively, these data suggest that a high-mannose restricted *N*-glycome, achieved in the *Mgat1*^*Δ/Δ*^ model, has a drastic influence on T-cell development, precluding the selection of peripheral T cells. This effect was not observed in the *Mgat2*^*Δ/Δ*^ model, suggesting that one GlcNAc antenna *N*-glycan is sufficient to guarantee developmental dynamics, highlighting a pathogenic role of high-mannose *N*-glycans.

### Mannosylated thymocytes exhibit deficient ß-selection

**Fig. 3:**
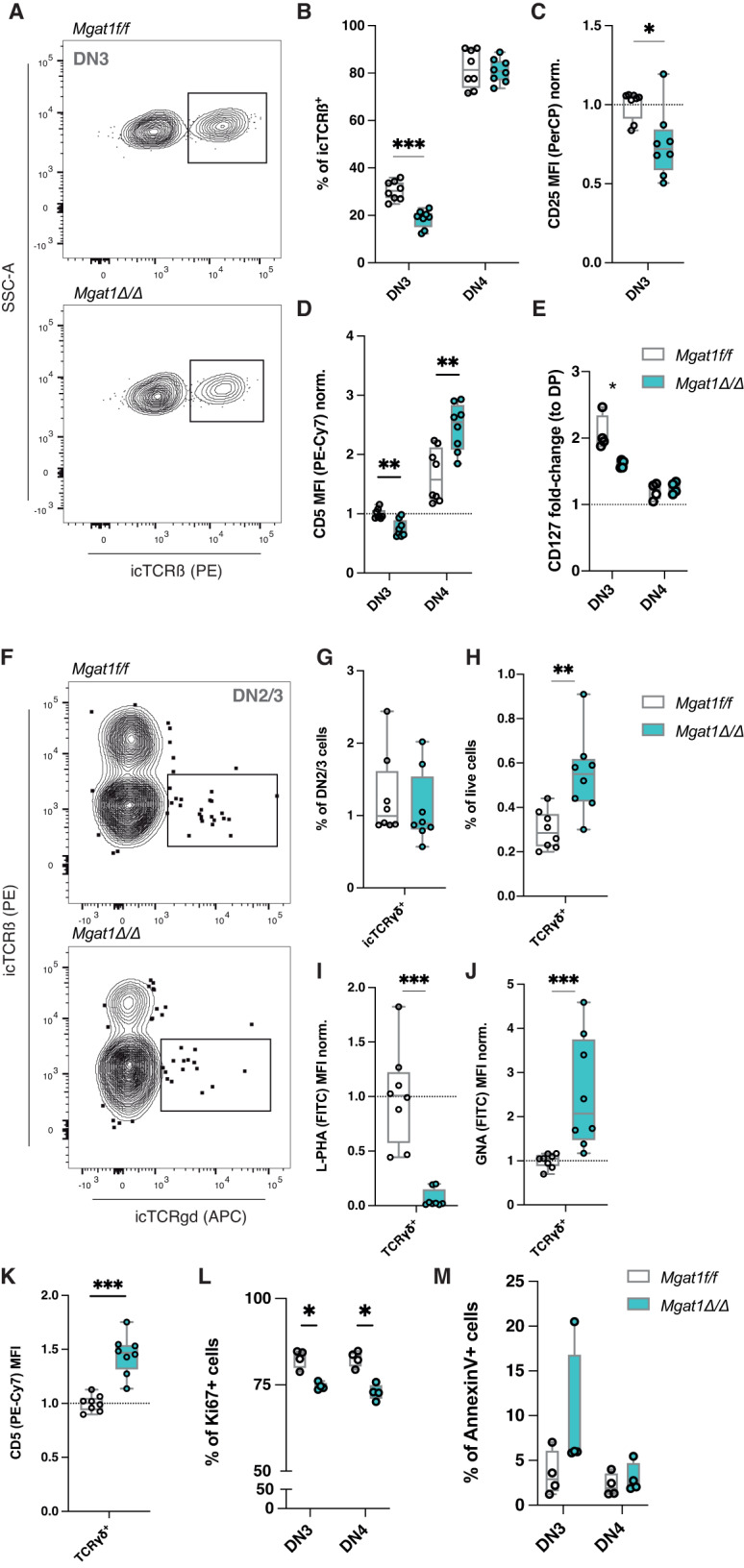
Lack of complex-branched N-glycans impairs ß-selection without affecting the determination of γδ T cells**. A** Gating strategy for the identification of ß-selected (icTCRß+) DN3 cells and (**B**) frequencies of those cells in DN3 and DN4 subsets in *Mgat1*^*f/f*^ (*N* = 8) and *Mgat1*^*Δ/Δ*^ mice (*N* = 8). **C** Quantification of CD25 surface levels (MFI) in the DN3 subset normalized to the mean of *Mgat1*^*f/f*^ DN3 levels. **D** Quantification of CD5 and (**E**) CD127 surface levels (MFI) in DN3 and DN4 thymocytes normalized to the mean of *Mgat1*^*f/f*^ DN3 levels and CD127 DP levels, respectively. **F** Gating strategy for the identification of icTCRγδ + cells within the DN2/3 subset and (**G**) frequencies in *Mgat1*^*f/f*^ (*N* = 8) and *Mgat1*^*Δ/Δ*^ mice (*N* = 8). **H** Frequency of total thymic mature TCRγδ + cells within live cells. **I** Levels of L-PHA and (**J**) GNA binding levels in thymic TCRγδ + cells normalized to the mean *Mgat1*^*f/f*^ levels. **K** CD5 surface levels (MFI) of TCRγδ + cells, normalized to the mean *Mgat1*^*f/f*^ levels. **L** Quantification of Ki67+ cell frequencies within the DN3 and DN4 populations. **M** Quantification of apoptotic cells (Annexin V + ) within the DN3 and DN4 populations. Each dot represents one mouse. Mann‒Whitney *t*-test, *p* value * < 0.05, ** < 0.005 and *** < 0.001

Given the alterations in the frequency of DN cells imposed by *Mgat1* deficiency, particularly the decrease in DN3 and the increase in DN4 subpopulations, we sought to investigate the impact of a high-mannose restricted *N*-glycome on ß-selection. This key checkpoint translates the functionality of the newly generated ß-chain TCRs, a product of *Tcrb* gene rearrangements [1, 2]. ß-Selected DN3 cells begin to express pre-TCR complexes, detected by the expression of intracellular (ic)TCRß chain [1, 20]. To evaluate the role of high-mannose *N*-glycans in ß-selection, we first determined the frequency of DN3 cells bearing icTCRß. We observed a significant decrease in icTCRß+ DN3 cells but not DN4 cells in the *Mgat1*^*Δ/Δ*^ model (Fig. 3A, B), suggesting that clonal expansion of ß-selected thymocytes was not compromised but rather that their generation was. Moreover, in the absence of *Mgat1*, decreased CD25 surface expression was detected in DN3 cells (Fig. 3C), suggesting ß-selection defects [21]. Pre-TCR signaling, responsible for the induction of DN3 proliferation and DN4 transition, can be detected by proxy CD5 expression [22]. In the *Mgat1*^*Δ/Δ*^ model, we found a decrease in CD5 in DN3 cells but not in DN4 cells (Fig. 3D). Thus, high-mannose restricted *N*-glycomes interrupt the generation of ß-selected thymocytes but do not alter their clonal expansion. Moreover, as the disruption of ß1,6-GlcNAc branching *N*-glycans promotes mature TCR clustering and T-cell hyperactivation [10, 14], the clustering of pre-TCR complexes could play a role, which deserves further investigation. As IL-7 signaling is one of the key regulators of ß-selection [1, 2, 20], we evaluated IL-7Rα (CD127) expression in DN3 cells and found a decrease in *Mgat1*^*Δ/Δ*^ thymocytes (Fig. 3E), which agrees with the perturbation of ß-selection. In accordance with previous results, in the presence of mono-antennary *N*-glycans on *Mgat2*^*Δ/Δ*^ mice, we detected no differences in the parameters regarding ß-selection (Supplementary Fig. 3A–E). As ß-selection leads to DN3 cellular proliferation, followed by DN4 expansion, we evaluated the frequency of proliferating cells in these subsets. Interestingly, *Mgat1*^*Δ/Δ*^ DN3 thymocytes displayed lower frequencies of proliferating cells than their littermate controls, which is in line with previous results, suggesting a ß-selection deficiency in these mice (Fig. 3L). Moreover, we observed a tendency for increased apoptotic DN3 cells (Fig. 3M). These differences were not detected in *Mgat2*^*Δ/Δ*^ mice (Supplementary Fig. 3L and M). Thus, these results pinpoint the hampering of ß-selection and pre-TCR signaling by high-mannose *N*-glycans, thus revealing the role of *N*-glycan complexity in this developmental checkpoint.

### High-mannose *N*-glycans bias T-cell development, favoring γδT-cell development

T-cell commitment toward the γδT-cell lineage occurs early at the DN2 and DN3 stages, where *Tcrg* and *Tcrd* may also be targeted for genetic rearrangements [3]. The precise mechanisms underlying γδ lineage commitment have not yet been fully clarified [23]. Since the absence of *Mgat1* leads to deficient generation of DN3 ß-selected cells (Fig. 3A–E), we analyzed the presence of DN2/3 cells bearing intracellular (ic)TCRγδ, but no differences were detected in either *Mgat1-*deficient (Fig. 3F and G) or *Mgat2-*deficient thymocytes (Supplementary Fig. 3F and G). However, an increased proportion of thymic mature γδT cells was observed in both glycoengineered models (Fig. 3H; Supplementary Fig. 3H), although there were no changes in the absolute numbers of these cells (Fig. 2I). These cells were shown to display an absence of L-PHA binding (Fig. 3I; Supplementary Fig. 3I) and increased mannose expression (GNA binding) in both models (Fig. 3J; Supplementary Fig. 3J). When we evaluated the TCR-mediated activation status of these mannosylated cells, we observed increased CD5 in thymic γδT cells of *Mgat1*^*Δ/Δ*^ (Fig. 3K) but not in *Mgat2*-deficient mice (Supplementary Fig. 3K), suggesting higher TCR signaling in *Mgat1*-deficient γδT cells, which has been associated with the promotion of development of this T-cell subset [23]. Altogether, the regulation of γδTCR signaling intensities by *N*-glycans represents a potential mediator in the development of those cells.

Our results suggest that high-mannose *N*-glycomes in DN2/3 cells favor γδT lineage commitment and should play a major role in the regulation of their activation, as in peripheral αβTCR T cells [4].

### High-mannose *N*-glycans disturb the TCR repertoire diversity

Previous evidence has shown that *N*-glycans regulate positive and negative DP selection in a *Lck*^Cre/+^*Mgat1*^f/f^ background, where ~70% of DPs were defective in complex-branched *N*-glycans [13]. In our *Mgat1*^*Δ/Δ*^ model, in which >90% of DP cells are deficient in complex-branched *N*-glycans, the DP-selection effect was validated. We analyzed the differential expression of the TCRß chain and CD69 (Supplementary Fig. 4A and D) to identify the thymocyte populations undergoing selection. As expected, *Mgat1* deficiency caused a significant increase in the frequency of TCRß^−^CD69^−^ cells (Supplementary Fig. 4B), with higher apoptosis levels (Supplementary Fig. 4C), indicating increased death by neglect. Accordingly, a decrease in positively selected cells, that is, TCRß^hi^CD69^hi^, was observed (Supplementary Fig. 4B). Finally, postselection thymocytes, namely, TCRß^hi^CD69^−^ were reduced in *Mgat1*^*Δ/Δ*^ mice (Supplementary Fig. 4B), suggesting a more pronounced negative selection. Again, these differences were not observed in the *Mgat2*^*Δ/Δ*^ mice, as the populations of DP-selection as well as the apoptosis levels were similar to those of their control counterparts (Supplementary Fig. 4D-F).

**Fig. 4:**
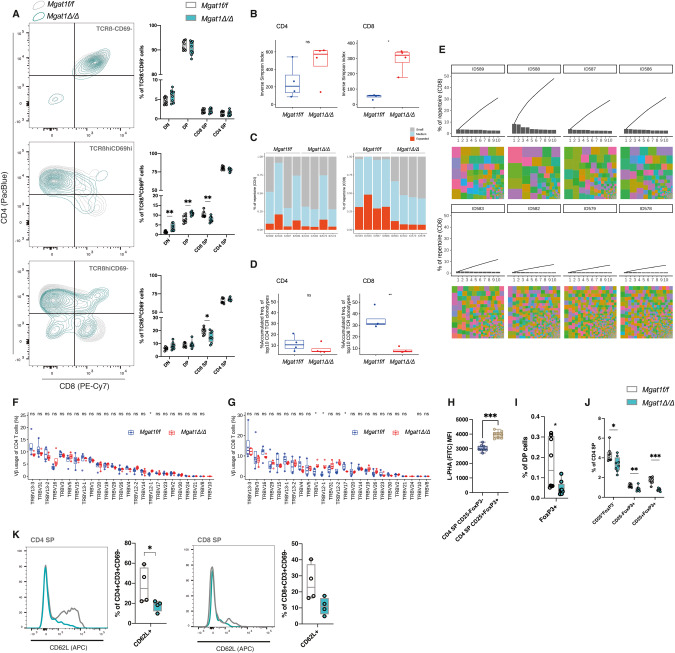
TCR-selection is impaired in the high-mannose restricted *N*-glycome, with severe effects on TCR repertoire diversity and thymic Treg generation**. A** Distribution of each selection subset, preselection, postpositive selection and postnegative selection thymocytes, according to the CD4 and CD8 expression levels, and quantification in *Mgat1*^*f/f*^ (*N* = 8) and *Mgat1*^*Δ/Δ*^ mice (*N* = 8). **B** Inverse Sympson index of CD4 SP and CD8 SP TCRvß repertoires. **C** Visualization of clone proportions: most frequent 10 (top 10) – expanded clones; 11–100th most frequent – medium clones; other clones – small clones. **D** Top 10 clone accumulative frequencies of CD4 SP and CD8 SP. **E** Top 10 clone frequencies (bars) and treemap visualization showing clone distributions, where each colored box represents the abundance of each clone by its size, colored by V-genes (each color is assigned randomly and does not match between the samples). **F** Distribution according to each detected Vß region in CD4 SP and (**G**) CD8 SP mature thymocytes. **H** Levels of L-PHA binding (MFI) in wild-type thymic CD4 SP CD25-FOXP3- and Treg populations. **I** Quantification of the frequency of FoxP3+ cells in the DP population. **J** Frequencies of subsets within CD4 SP thymocytes. **K** Identification of CD62L^+^ cells in mature CD4 SP and CD8 SP cells in *Mgat1* WT (*N* = 4) and *Mgat1* cKO (*N* = 4) and *Mgat2* WT (*N* = 4) and *Mgat2* cKO (*N* = 4) mice. Each dot represents one mouse. Mann‒Whitney *t-*test, *p* value * < 0.05, ** < 0.005 and *** < 0.001

Once positively selected, DP thymocytes undergo CD8/CD4 lineage choice, a process regulated by TCR signaling dynamics [24]. We then analyzed CD8/CD4 lineage commitment by analyzing the relative distributions of the three populations described above according to CD8/CD4 expression (Fig. 4A). Interestingly, positively selected cells were differentially distributed according to their CD8 and CD4 surface expression in both models (Fig. 4A), with the CD8 SP quadrant underrepresented in postnegative selection cells in the *Mgat1*-deficiency model (Fig. 4A), with no major differences in *Mgat2*^*Δ/Δ*^ mice (Supplementary Fig. 4I). These results indicate a role for *N*-glycans in CD4/CD8 lineage commitment. In addition, with positive selection, cells proliferate until they reach the negative selection checkpoint. Interestingly, we observed lower levels of Ki67+ cells within the mature CD4 SP and CD8 SP subsets in *Mgat1*^*Δ/Δ*^ mice (Supplementary Fig. 4G) and no differences in *Mgat2*^*Δ/Δ*^ mice compared to their littermate controls (Supplementary Fig. 4H).

One of the most important events occurring during TCR selection is the generation of a functional TCR repertoire. Given the impact of *N*-glycans on TCR selection, we evaluated whether the selection defects observed in *Mgat1*^*Δ/Δ*^ mice could generate altered TCR diversity. For this, we sequenced the TCRvß regions in the mature SP subsets. Notably, in *Mgat1*^*Δ/Δ*^ mice, we found a significant impact on the diversity of the TCRvß repertoire in mature CD8 SP (Fig. 4B). In the *Mgat1*-deficient model, we observed increased TCRvß diversity in CD8 SP, which was accompanied by an increase in low-represented TCR clones and a decrease in expanded ones (Fig. 4C), which is evident in the accumulated frequency of the top 10 TCR clones in CD8 SP and not in CD4 SP (Fig. 4D and E). The analysis of specific TCRvß regions showed differential usage of specific Vß regions in CD4 SP and CD8 SP (Fig. 4F and 4G). The sequencing reads were not significantly different between the genotypes (Supplementary Fig. 4J). No major differences in the TCR repertoire were observed for *Mgat2*^*Δ/Δ*^ thymocytes using a panel of TCRvß-targeting antibodies (Supplementary Fig. 4K and L). These results further reveal the central role of *N*-glycan structures in DP selection, namely, in lineage commitment and TCR repertoire diversity.

### High-mannose *N*-glycans hamper regulatory T-cell development

The ability of complex-branched *N*-glycans to control the thresholds of TCR positive and negative selection prompted us to raise the question of whether these structures could influence the thymic development of T regulatory cells (Tregs). Naturally occurring Tregs (nTregs) are generated in the thymus, comprising a set of CD4 SP cells with high levels of activation close to the threshold of negative selection [25]. In fact, the upregulation of complex *N*-glycans of thymic Tregs, when compared to conventional CD4 SP thymocytes, in wild-type mice (Fig. 4H) showcases a dynamic glycoprofile alteration. In this work, and previous ones [13], it is shown that negative selection is promoted in *Mgat1*-deficient DPs (Supplementary Fig. 4A–C), so we argued that Treg generation would be impaired. The initial precursors of Tregs are DP cells, which show considerable expression of the FoxP3 transcription factor [25]. Interestingly, this population was decreased in an *Mgat1*-deficient scenario (Fig. 4I), whereas no differences were found for *Mgat2* deficiency (Supplementary Fig. 4M). As mature Tregs may arise from CD4^+^CD25^+^FOXP3^+^ and CD4^+^CD25^−^FOXP3^lo^ progenitors, we evaluated the presence of these two subsets in the thymic compartment. We detected a lower frequency of both populations in *Mgat1*^*Δ/Δ*^ mice (Fig. 4J), and no differences were found in *Mgat2*^*Δ/Δ*^ mice (Supplementary Fig. 4N). Moreover, the absolute numbers of thymic Tregs were severely diminished in *Mgat1*^*Δ/Δ*^ mice compared to their wild-type controls (Fig. 2I). Thus, our results highlight an impact of high-mannose *N*-glycans on thymic Treg generation, and this effect is not observed in the presence of hyperelongated hybrid *N*-glycans.

### High-mannose *N*-glycans impair thymic egress of mature thymocytes

The final step in T-cell development is thymic egress, where thymocytes that surmounted negative selection upregulate CD62L expression and leave the thymus [26]. The presence of mature and selected CD4 and CD8 SP thymocytes in both murine models was assessed, and the relative expression of CD3 and CD69 was used to detect postselection cells. A remarkable decrease in the frequency of CD62L^+^ cells was observed in postselection CD4 SP in the *Mgat1*^*Δ/Δ*^ mice, which was fully compensated for in *Mgat2*^*Δ/Δ*^ mice, whereas no differences in the CD8 SP subset (Fig. 4K and Supplementary Fig. 4O) were detected, although a trend toward a decrease in the CD8 SP subset could be observed in the absence of branched *N*-glycans. Thus, as with the previous developmental checkpoints, thymic egress is markedly compromised when complex *N*-glycans are absent.

### Mannosylated thymocytes promote peripheral γδT-cell activity and the immune response

**Fig. 5:**
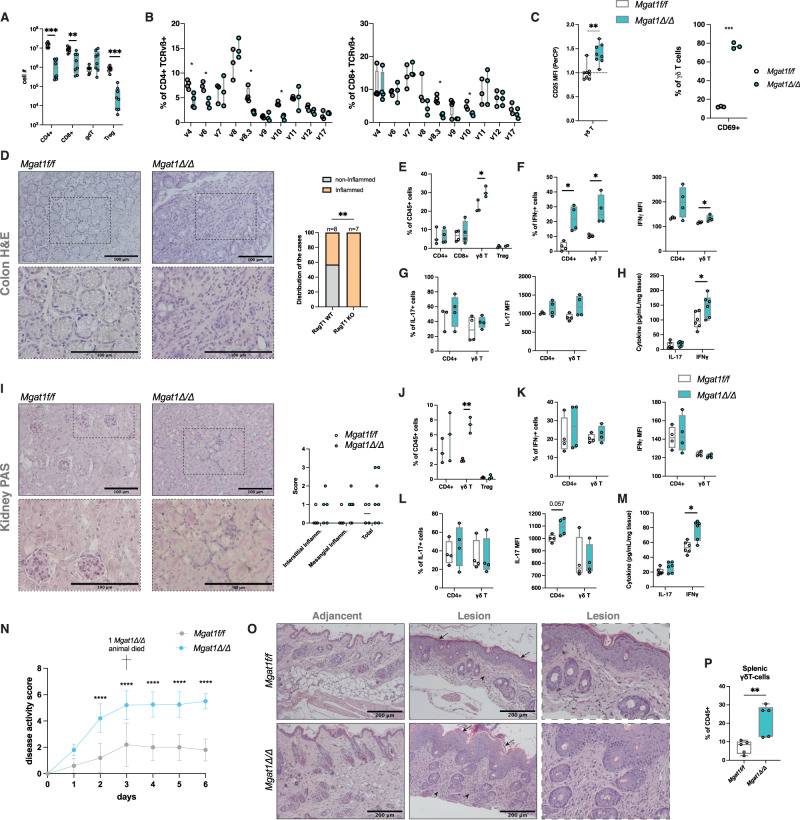
Impairments in T-cell development induced by the absence of complex N-glycans lead to peripheral alterations in homeostasis**. A** Splenic T-cell numbers in *Mgat1*^*f/f*^ (*N* = 8) and *Mgat1*^*Δ/Δ*^ (*N* = 8). **B** Screening of TCRvß+ expressing cells in splenic mature CD4+ (right) and CD8 + T cells (right) in *Mgat1*^*f/f*^ (*N* = 4) and *Mgat1*^*Δ/Δ*^ (*N* = 4). **C** Levels of CD25 surface expression (MFI) in splenic γδ T cells, normalized to the mean *Mgat1*^*f/f*^ levels, and the quantification of CD69+ cells within this subset. **D** H&E staining of colon sections at ×20 (top) and ×40 magnification (bottom) and specimen distribution according to overall detection of inflammation cues (right). Scale bars indicate 100 µm. **E** T-cell population frequencies within CD45+ cells isolated from colon tissues. **F** Frequencies of IFNγ-producing cells within CD4+ and γδ T cells and IFNγ expression (MFI) in the same subsets. **G** Frequencies of IL-17-producing cells within CD4+ and γδ T cells and IL-17 expression (MFI) in the same subsets. **H** IL-17 and IFNγ concentrations in colon explant culture supernatants normalized to tissue weight. **I** PAS staining of colon sections at ×20 (top) and ×40 magnification (bottom) and scores of interstitial and mesangial inflammation (right). Scale bars indicate 100 µm. **J** T-cell population frequencies within CD45+ cells isolated from kidney tissues. **K** Frequencies of IFNγ-producing cells within CD4+ and γδT cells and IFNγ expression (MFI) in the same subsets. **L** Frequencies of IL-17-producing cells within CD4+ and γδT cells and IL-17 expression (MFI) in the same subsets. **M** IL-17 and IFNγ concentrations in colon explant culture supernatants normalized to tissue weight. **N** Disease activity score for the imiquimod-induced psoriasis model for *Mgat1*^*f/f*^ (*N* = 5 females) and *Mgat1*^*Δ/Δ*^ (*N* = 5 females) mice. **O** Representative histological analysis of the skin of these mice from lesion-adjacent and lesion tissue. Arrows highlight epithelial thickening, and arrowheads highlight immune infiltrate. **P** Quantification of splenic γδT cells on the final day of the experiment. Each dot represents one mouse. Mann‒Whitney *t*-test, *p* value * < 0.05, ** < 0.005 and *** < 0.001

Given the number of defects observed in T-cell development upon *Mgat1* deficiency, we further explored the biological consequences of this abnormal expression of mannosylated glycans within the peripheral compartment in both models, as a lack of complex *N*-glycans has been associated with pathogenic scenarios [10, 11, 27, 28]. In fact, we observed decreased L-PHA binding in circulating T-cells from inflammatory bowel disease patients, which supports the biological impact of changes in the T-cell glycoprofile on disease susceptibility (Supplementary Fig. 1A). We observed a drastic reduction in splenic CD4^+^ and CD8^+^ T-cell numbers in *Mgat1*^*Δ/Δ*^ mice (Fig. 5A), with no differences observed in *Mgat2*^*Δ/Δ*^ mice (Supplementary Fig. 5B), as was reported by others [13]. We then evaluated the impact on the TCRvß repertoire in splenic CD4^+^ and CD8^+^ T-cells and found critical differences in *Mgat1*^*Δ/Δ*^ mice (Fig. 5B), suggesting a significant reduction in the diversity of peripheral TCRvß repertoires in comparison with that in control mice, with no alterations in *Mgat2*^*Δ/Δ*^ mice (Supplementary Fig. 5C). Interestingly, in *Mgat1*^*Δ/Δ*^ glycoengineered mice, we observed no alterations in the total numbers of splenic γδT cells (Fig. 5A), showcasing a resistance of this subset to a pathogenic high-mannose restricted *N*-glycome. In fact, in agreement with the increased activation status of mature thymic γδT-cells (Fig. 3K), *Mgat1*-deficient splenic γδT-cells showed increased levels of CD25 expression, and we observed increased frequencies of CD69+ cells within this subset related to cellular activation (Fig. 5C). Neither of these differences were observed in *Mgat2*^*Δ/Δ*^ mice (Supplementary Fig. 5D). To our knowledge, this is the first report demonstrating the impact of the mannosylated *N*-glycan pathway in promoting peripheral γδT-cell activity.

Given this effect on the activity of γδT-cells at the periphery, we next explored the pathophysiologic consequences, at baseline, of these glycoengineered murine models. The histological analysis of colon sections of *Mgat1*-pro and -deficient models showed increased immune infiltrates in the latter group, related to inflammation (Fig. 5D), which was not observed in the *Mgat2*^*Δ/Δ*^ model (Supplementary Fig. 5E). Moreover, immunohistochemistry analysis of colon sections showed higher CD3^+^ immune infiltrates (Supplementary Fig. 5O). In addition, flow cytometry analysis of CD45^+^ cells from the colonic tissue revealed increased γδT-cell frequencies (Fig. 5E) for *Mgat1*^*Δ/Δ*^ mice, which was accompanied by increased IFNγ-producing CD4+ and γδT cells (Fig. 5F). No differences in the frequency of IL-17-producing CD4+ and γδT cells or in IL-17 expression (Fig. 5G) were observed. Moreover, and in agreement with the flow cytometry data, colon explants from *Mgat1*^*Δ/Δ*^ mice released significantly more IFNγ (Fig. 5H). No differences in the serum levels of these two cytokines were observed in *Mgat1*^*Δ/Δ*^ mice (Supplementary Fig. 5R). In *Mgat2*^*Δ/Δ*^ mice, although the analysis of colon sections revealed some degree of inflammation (Supplementary Fig. 5E), no significant differences were detected in the analysis of the immune infiltrates, IL-17/IFNγ-producing cells or explant cytokine release (Supplementary Fig. 5F–I). The analysis of fecal calprotectin levels, commonly used in the clinic as a marker of intestinal inflammation [29], revealed a slight tendency to increase in *Mgat1*^*Δ/Δ*^ in comparison with controls, which was absent in *Mgat2*^*Δ/Δ*^ (Supplementary Fig. 5P and Supplementary Fig. 5Q).

Having established colonic features of spontaneous inflammation in *Mgat1*^*Δ/Δ*^ that were compensated for in *Mgat2*^*Δ/Δ*^, we decided to analyze other putative target organs, such as the kidney, which is commonly affected in the context of chronic inflammation and autoimmunity, in a process that can be mediated by abnormal glycosylation [30]. The analysis of both the interstitial and mesangial compartments of the renal parenchyma showed increased inflammatory signs in *Mgat1*^*Δ/Δ*^ mice (Fig. 5I). Similar to the colon scenario, we observed increased γδT-cell kidney infiltration (Fig. 5J), and although no differences were seen in the frequencies of IFNγ-producing γδT-cells (Fig. 5K), an increased release of IFNγ in kidney explants from *Mgat1*^*Δ/Δ*^ mice was seen (Fig. 5M). Regarding IL-17-producing cells, no differences were found (Fig. 5L). No significant differences were observed in the *Mgat2*^*Δ/Δ*^ glycoengineered mice (Supplementary Fig. 5J–N). Together, these results showed phenotypic consequences of the restriction of high-mannose *N*-glycomes in thymocytes, highlighting the pathogenic role of these structures in T-cells and revealing that γδT-cells are potential drivers of spontaneous inflammation.

To further understand the immunological responses of the glycoengineered models, we tested an infection model. Both *Mgat1*^*Δ/Δ*^ and *Mgat2*^*Δ/Δ*^ mice were challenged with the obligate intracellular protozoan *N. caninum*, as resistance to this parasite was previously shown to be highly T-cell dependent [18]. Our results showed that *Mgat1*^*Δ/Δ*^ mice exhibited increased parasite colonization in the key organs analyzed, suggesting a tendency for increased susceptibility to infection when compared to their wild-type counterparts (Supplementary Fig. 5S), which was not observed in *Mgat2*^*Δ/Δ*^ mice (Supplementary Fig. 5U). Since *N. caninum*-related immune responses are highly dependent on an intact Th1 response [18], we further analyzed the immunophenotype of the splenocytes of infected mice and observed an expansion in both CD4^+^ and CD8^+^ T-cell frequencies and numbers in *Mgat1*^*Δ/Δ*^ mice compared to steady-state mice (Supplementary Fig. 5T), which presented a higher frequency of CD69^+^ cells in CD4^+^ T cells upon infection, which agrees with the role of complex *N*-glycans in TCR clustering. Moreover, we detected negligible levels of naïve T cells (CD62L^+^CD44^−^) in the spleens of these mice (Supplementary Fig. 5T). These results support an increased proinflammatory response in *Mgat1*^*Δ/Δ*^ mice that, together with the lowered threshold for T-cell activation, may explain the increased susceptibility to infection. Moreover, given the high levels of γδT-cell activation observed in *Mgat1*^Δ/Δ^ mice and to further understand the consequences of *N*-glycome restriction to high-mannose glycans in disease susceptibility, we employed the imiquimod-induced psoriasis model, which is known to be a γδT-cell-driven inflammatory disease model. Interestingly, the *Mgat1*-deficient model displayed a statistically significant increased susceptibility to disease starting at Day 2 of imiquimod application, which was maintained throughout the assay (Fig. 5N). Histological analysis of skin tissue revealed increased epidermal thickness with high levels of immune infiltrate in *Mgat1*^Δ/Δ^ mice compared to littermate controls (Fig. 5O). Finally, we detected an increased γδT-cell population in the spleen of *Mgat1*^Δ/Δ^ mice, indicating a clear γδT-cell-driven inflammatory response (Fig. 5P).

Altogether, our results show that an early thymocyte high-mannose restricted *N*-glycome creates a pathogenic glycophenotype characterized by abnormal expression of mannosylated *N*-glycans on T-cells, which has profound central and peripheral immune consequences by conferring increased susceptibility to infection, chronic inflammation and autoimmunity. Hyperelongation of *N*-glycans efficiently rescues this T-cell phenotype and prevents higher susceptibility to disease development.

## Discussion

T-cell development is a key biological process that ensures a functional and diverse TCR repertoire of T cells, enabling robust mechanisms of immune protection and tolerance. The discovery of cellular developmental mediators of thymocyte maturation has received attention since the 1960s, shaping modern immunology [31]. However, despite considerable progress, the function of cell-specific *N*-glycans in early T-cell lineage commitment and selection and their relevance in T-cell-mediated immunopathology have been largely unexplored. Here, we present a comprehensive characterization of the glycocalyx composition of both human and murine thymocytes, demonstrating the existence of unique glycosylation signatures that distinguish each developmental stage. In fact, the analysis of the thymocyte population glycome highlighted high *N*-glycan complexity and diversity across different thymocyte stages, namely, high levels of poly-LacNAc structures in immature subsets and elevation of terminal α2,6-sialylation in mature SP populations, both accompanied by a considerable presence of complex ß1,6-GlcNAc branching. Moreover, the overall low levels of high-mannose structures suggest *N*-glycan complexity as a *sine-qua-non* feature for functional developmental processes associated with homeostasis. In addition, single-cell RNA sequencing analysis further revealed that the glycotranscriptomic profile of selected glycogenes in thymocytes is developmentally regulated, highlighting the biological relevance of the thymocyte glycosignature in both human and murine T-cell development. In fact, we have previously reported altered T-cell glycoprofiles in several disease scenarios related to activation, which highlights the regulatory power of glycans in determining T-cell state identities [4, 28]. This manuscript is the first, to our knowledge, to assess with great depth the glycoprofile of human thymocytes. In addition, for murine thymocytes, this is the first report, to our knowledge, of a combined panel of lectin analyses of T-cell development.

To model the expression of *N*-glycans in thymocytes, we generated unique glycoengineered murine models with conditional ablation of branched *N*-glycan structures in T-cells through *Rag1*^Cre^-mediated disruption of *Mgat1* and *Mgat2* in DN2 thymocytes. A deficiency in the synthesis of complex-branched *N*-glycans and the consequent restriction of high-mannose *N*-glycan presence, modeled in *Mgat1*^*Δ/Δ*^ mice, resulted in an increase in the percentage of DN cells relative to total thymocytes that was associated with an impairment in ß-selection. In total DN3 cells, we showed decreased pre-TCR signaling in the absence of *Mgat1*, accompanied by decreased DN3 proliferation. Moreover, decreased expression of IL-7Rα may also contribute to impaired ß-selection and DN3 survival. Interestingly, IL-7Rα has 3 conserved *N*-glycosylation sites in mice and 6 in humans. The high-mannose glycans in *Mgat1*^*Δ/Δ*^ mice in this receptor may influence its dynamics, such as IL-7 affinity [32] or endocytosis, which deserves further study. As *N*-glycans control αßTCR thresholds, the same regulatory effect might be extrapolated to γδTCRs, promoting sustained signaling to γδT cells (in the *Mgat1*-deficient model) and favoring the development of this subset [23]. To our knowledge, this is the first report on the role of mannosylated *N*-glycans in promoting γδT-cell development, suggesting the critical role of *N*-glycan structures in the regulation of T-cell subset activity and function.

DP thymocytes are bipotential progenitors for the CD4^+^ and CD8^+^ T-cell lineages during positive selection. We found that the presence of only mannosylated structures in cells that have been positively selected was associated with an increased percentage of cells in the DN and DP quadrants, suggesting enhanced coreceptor internalization/endocytosis, as previously observed in other settings [13], which altered the fate of DP cells to the CD4 SP and CD8 SP lineages. Importantly, our results further identify an additional and novel role of *N*-glycans in TCR selection, demonstrating that high-mannose *N*-glycomes significantly alter the diversity of TCRvß variants in mature SP cells, specifically the lack of expanded TCRvß clones. This impact on TCR diversity should be due to deficient ß-selection, impaired positive selection and the promotion of negative selection in *Mgat1*^*Δ/Δ*^ mice. In addition, we found that thymic Treg generation is also compromised in the absence of complex-branched *N*-glycan structures. Impairment in the generation of nTregs could be explained by the reduction in the TCR affinity threshold for negative selection imposed by the absence of *N*-glycan branching [13]. Finally, CD62L-expressing mature CD4 SP thymocytes in *Mgat1*^*Δ/Δ*^ were diminished, suggesting an impairment in egression, which we cannot exclude as being influenced by the previous selection processes. Altogether, our results demonstrated that thymocyte glycocalyxes are developmentally and dynamically regulated and that high-mannose *N*-glycomes do not ensure proper transitions between developmental stages or regulate central selection and commitment processes. Elegant studies from Demetriou’s group on the contribution of *N*-glycans in TCR selection showed its impact on the regulation of the thresholds for positive and negative selection [13]. Our work, targeting *Mgat1* at earlier stages of T-cell development (*Rag1*^Cre^), has illuminated the contribution of *N*-glycans, specifically highly mannosylated *N*-glycan structures, in several critical developmental processes, including lineage commitment, TCR repertoire determination, thymic Treg generation, γδT-cell development and thymic egress, as well as the phenotypic consequences in terms of disease susceptibility.

In this study, we showed that GlcNAc-branching of *N*-glycans is a *sine-qua-non* condition for appropriate T-cell development, as highlighted by the fact that *Mgat2*^*Δ/Δ*^ mice, proficient in synthesizing mono-antennary *N*-glycan structures in T cells, do not display major developmental defects, exhibiting normal ß-selection, production of diverse TCR repertoires, normal generation of Tregs and thymic egress, as well as γδT-cell development. This finding suggests that hyperelongated mono-antennary *N*-glycans are key glycan determinants that are essential in T-cell development and able to rescue the majority of the developmental processes that are markedly impaired in *Mgat1*-deficient mice. The synthesis of a mono-antennary *N*-glycan structure appears to create a compensatory mechanism that allows glycan compensatory elongation, which is required for galectin-targeting and terminal sialylation, and is thus essential to feed a proper T-cell selection program. These glycosylation compensatory pathways have been described in other physiopathological settings [19].

The phenotypic impact of such a drastic impairment on T-cell development was demonstrated in *Mgat1*^*Δ/Δ*^ mice, which exhibited an increased susceptibility, at baseline, to inflammation and infection and in an experimental psoriasis model. A central deficiency of complex-branched *N*-glycans on T-cells results in markedly reduced splenic αβT-cells. Moreover, we detected a colonic and kidney inflammation phenotype, mainly involving increased IFNγ presence/production, in *Mgat1*^*Δ/Δ*^ mice. The absence of complex *N*-glycans in T-cells has been shown to decrease their activation thresholds [4], which agrees with the inflammatory cues we observed. Moreover, the tendency for increased susceptibility to *N. caninum* infection, with increased T-effector polarization, agrees with the overall phenotype of increased inflammation. Finally, together with the increased susceptibility of *Mgat1*^Δ/Δ^ mice to inflammatory/infectious diseases and the increase in splenic γδT-cells, this set of results reveals a previously unknown pathogenic feature of mannosylated *N*-glycans on thymocytes, that is, their influence on the activity status of γδT-cell-associated inflammation.

The role of the immune system in congenital disorders of glycosylation (CDG) is receiving some attention from the clinical and scientific community [33]. One of the aspects that those works highlights is the pathogenic role of mannosylated N-glycans (also aberrantly present in several forms of CDGs) in T-cell development, with peripheral consequences, namely, in inflammatory cues. Altogether, the evidence on the role of glycans in immune development highlights the need for further studies of CDG patients.

Taken together, this study unveils the regulatory power of branched *N*-glycans in T-cell development, highlighting their role as key determinants for immunity and as essential players in the control of ß-selection, Treg generation, TCR repertoire diversity and γδT-cell development. Our findings constitute novel features of thymocyte “identity” and function by revealing that branched *N*-glycans are essential structures of T-cells. We also reveal the implications of its deficiency giving rise to “pathogenic” mannose structures on T-cells that drive increased susceptibility to the development of major diseases, such as infection and inflammation.

## Supplementary information

Supplemental Figure legends and Table 1

Supplemental Figure 1

Supplemental Figure 2

Supplemental Figure 3

Supplemental Figure 4

Supplemental Figure 5

## Data Availability

FASTQ files of *Trb* sequences are available under the SRA accession code PRJNA961050.
