# Supplementary material for: Mannosylated glycans impair normal T-cell development by reprogramming commitment and repertoire diversity
Source: Cell Mol Immunol. 2023 Jun 21;20(8):955–68. doi: 10.1038/s41423-023-01052-7 (PMC10387478; doi:10.1038/s41423-023-01052-7)
Supplement: Supplementary file 1 — Supplemental Figure legends and Table 1 [file 41423_2023_1052_MOESM1_ESM.docx]

**Supplementary Figures legends**

**Sup. Fig. 1: (A)** Gating strategy for human thymocyte subsets identification. On top of dot plots the parent gate is detailed. **(B)** Unsupervised flow cytometry analysis of human thymocytes using tSNE, combining the data from 4 human thymi, with the main thymocyte populations annotated manually by the gating strategy defined in (A). Colormap for L-PHA and SNA (right) binding levels in the same tSNE representation. Colorbar indicates the levels of binding. **(C)** Uniform manifold approximation and projection (UMAP) dimensionality reduction visualization of the human thymocyte single-cell RNA sequencing dataset used (16). **(D)** Analysis of key glycogene expression in a human thymocyte single-cell RNA sequencing dataset (16). **(E)** Gating strategy for murine thymocyte subsets identification. On top of dot plots the parent gate is detailed. **(F)** Uniform manifold approximation and projection (UMAP) dimensionality reduction visualization of the murine human thymocyte single-cell RNA sequencing dataset used (16). **(G)** Analysis of key glycogene expression in a murine thymocyte single-cell RNA sequencing dataset (16).

**Sup. Fig. 2: (A)** Thymocyte populations discrimination in *Mgat2^f/f^* (N = 8, 6-8 weeks old) and *Mgat2^Δ/Δ^* (N = 8, 6-8 weeks old). **(B)** Frequencies of DN (CD4-CD8-), DP (CD4+CD8+), CD8 SP (CD4-CD8+) and CD4 SP (CD4+CD8-) thymocyte subsets, within live cells. **(C)** Frequencies of DN1 (CD44+CD25-), DN2 (CD44+CD25+), DN3 (CD44-CD25+) and DN4 (CD44-CD25-) subsets, within the total DN population. **(D)** CD8 SP and CD4 SP subset frequencies: ISP8 (CD8+CD24+CD3-), CD8 MSP (CD8+CD24int/-CD3+), CD4 ISP (CD4+CD24hiCD3lo/hi) and CD4 MSP (CD4+CD24loCD3hi), within live cells. Levels of lectin binding (MFI levels) normalized to indicated population (left) and representative histograms of lectin binding profiles in indicated thymocyte subsets, for L-PHA **(E)**, GNA **(F)**, LEL **(G)** and SNA **(H)**. **(I)** Surface expression (MFI) of the CD4 and **(J)** CD8 co-receptors in indicated populations, normalized to the value of DP cells. **(K)** Absolute numbers of thymocytes in the indicated subsets. **(L)** Surface expression (MFI) of the CD4 and **(M)** CD8 co-receptors in indicated populations, normalized to the value of DP cells.Each dot represents one mouse in all graphs. Mann-Whitney t-test, *p*-value *< 0.05, **< 0.005 and ***< 0.001.

**Sup. Fig. 3: (A)** Gating strategy for the identification of ß-selected (icTCRß+) DN3 cells and **(B)** frequencies of those cells in DN3 and DN4 subsets, in *Mgat2^f/f^* (N=8) and *Mgat2^Δ/Δ^* (N=8). **(C)** Quantification of CD25 surface levels (MFI) in the DN3 subset, normalized to the mean of *Mgat2^f/f^* DN3 levels. **(D)** Quantification of CD5 and **(E)** CD127 surface levels (MFI) in DN3 and DN4 thymocytes, normalized to the mean of *Mgat2^f/f^* DN3 levels, and CD127 DP levels, respectively. **(F)** Gating strategy for the identification of icTCRγδ+ cells within the DN2/3 subset and **(G)** frequencies, in *Mgat2^f/f^* (N=8) and *Mgat2^Δ/Δ^* (N=8).  **(H)** Frequency of total thymic mature TCRγδ+ cells within live cells. **(I)** Levels of L-PHA and **(J)** GNA binding levels, in thymic TCRγδ+ cells, normalized to the mean of *Mgat2^f/f^* levels. **(K)** CD5 surface levels (MFI) of TCRγδ+ cells, normalized to the mean of *Mgat2^f/f^* levels. **(L)** Quantification of Ki67+ cell frequencies within DN3 and DN4 populations. **(M)** Quantification of apoptotic cells (AnnexinV+) with DN3 and DN4 populations. Each dot represents one mouse. Mann-Whitney t-test, p-value *< 0.05, **< 0.005 and ***< 0.001.

**Sup. Fig. 4: (A)** and **(D)** Identification of pre-selection (TCRß^-/lo^CD69^-^), post-positive selection (TCRß^int/hi^CD69^int/hi^) and post-negative selection (TCRß^hi^CD69^-^) thymocytes in *Mgat1^f/f^* (N = 8) and *Mgat1^Δ/Δ^* (N = 8), and *Mgat2^f/f^* (N = 8) and *Mgat2^Δ/Δ^* (N = 8) mice, respectively. **(B)** and **(E)** Quantification of the frequencies of the populations identified in **(A)** and **(D)**. **(C)** and **(F)** Quantification of Annexin V+ cells within the thymocyte populations in *Mgat1^f/f^* (N = 4) and *Mgat1^Δ/Δ^* (N = 4), and *Mgat2^f/f^* (N = 4) and *Mgat2^Δ/Δ^* (N = 4) mice, respectively. **(G)** Quantification of Ki67+ cells within mature CD4 SP and CD8 SP in *Mgat1^f/f^* (N = 4) and *Mgat1^Δ/Δ^* (N = 4) and **(H)** *Mgat2^f/f^* (N = 4) and *Mgat2^Δ/Δ^* (N = 4) mice. **(I)** Distribution of each selection subset, pre-selection, post-positive selection and post-negative selection thymocytes, according to its CD4 and CD8 expression levels, and quantification in *Mgat2^f/f^* (N = 8) and *Mgat2^Δ/Δ^* (N = 8). **(J)** Quantification of the number of sequencing reads for the sorted CD4 SP and CD8 SP from *Mgat1^f/f^* (N = 4) and *Mgat1^Δ/Δ^* (N = 4). **(K)** Screen of TCRvß+ expressing cells, within thymic mature CD4 SP and **(L)** CD8 SP cells, in *Mgat2^f/f^* (N = 4) and *Mgat2^Δ/Δ^* (N = 4). **(M)** Quantification of the frequency of FoxP3+ cells in the DP population. **(N)** Frequencies of CD25+FOXP3-, CD25-FOXP3+ and CD25+FOXP3+ subsets with CD4 SP thymocytes. **(O)** Identification of CD62L^+^ cells in mature CD4 SP and CD8 SP, in *Mgat2^f/f^* (N = 4) and *Mgat2^Δ/Δ^* (N = 4) mice. Each dot represents one mouse. Mann-Whitney t-test, p-value *< 0.05, **< 0.005 and ***< 0.001.

**Sup. Fig. 5: (A)** Analysis of L-PHA binding to T-cells from PBMCs of inflammatory bowel disease patients (N=3) and healthy controls (N=3). **(B)** Splenic T-cell numbers cell numbers in *Mgat2^f/f^* (N = 8) and *Mgat2^Δ/Δ^* (N = 8). **(C)** Screen of TCRvß+ expressing cells, within splenic mature CD4+ (right) and CD8+ T-cells (right), in *Mgat2^f/f^* (N = 4) and *Mgat2^Δ/Δ^* (N = 4). **(D)** Levels of CD25 surface expression (MFI) in splenic γδ T-cells, normalized to the mean of *Mgat2* WT levels, and the quantification of CD69+ cells within this subset. **(E)** H&E staining of colon sections, at 20x (top) and 40x magnification (bottom), and specimen distribution according to overall detection of inflammation cues (right). Scale bars indicate 100 um. **(F)** T-cell population frequencies within CD45+ cells, isolated from colon tissues. **(G)** Frequencies of IFNγ-producing cells within CD4+ and γδ T-cells and IFNγ expression (MFI) in the same subsets. **(H)** Frequencies of IL-17-producing cells within CD4+ and γδ T-cells and IL-17 expression (MFI) in the same subsets. **(I)** IL-17 and IFNγ concentration in colon explant culture supernatants, normalized to tissue weight. **(J)** PAS staining of colon sections, at 20x (top) and 40x magnification (bottom), and scores of intersticial and mesangial inflammation (right). Scale bars indicate 100 um. **(K)** T-cell population frequencies within CD45+ cells, isolated from kidney tissues. **(L)** Frequencies of IFNγ-producing cells within CD4+ and γδ T-cells and IFNγ expression (MFI) in the same subsets. **(M)** Frequencies of IL-17-producing cells within CD4+ and γδ T-cells and IL-17 expression (MFI) in the same subsets. **(N)** IL-17 and IFNγ concentration in colon explant culture supernatants, normalized to tissue weight. **(O)** Immunohistochemistry staining for CD3+ cells in colon sections. **(P)** and **(Q)** Fecal calprotectin concentration analysis for *Mgat1^f/f^* (N = 5) and *Mgat1^Δ/Δ^* (N = 7), and *Mgat2^f/f^* (N = 5) and *Mgat2^Δ/Δ^* (N = 4). **(R)** Serum concentrations of IFNγ and IL-17. **(S)** *N. caninum* organ colonization determination, through the quantification of total parasite DNA in 1 mg of total host DNA, in *Mgat1^f/f^* (N = 3 - 4) and *Mgat1^Δ/Δ^* (N = 4). **(T)** T-cell frequencies of the spleens of infected mice and frequencies of CD69+ cells within CD4+ and CD8+ splenic T-cells. **(U)** *N. caninum* organ colonization determination, through the quantification of total parasite DNA in 1 mg of total host DNA, in *Mgat2^f/f^* (N = 3) and *Mgat2^Δ/Δ^* (N = 3). Each dot represents one mouse. Mann-Whitney t-test, p-value *< 0.05, **< 0.005 and ***< 0.001.

**Supplementary Table 1**

| REAGENT or RESOURCE | SOURCE | IDENTIFIER |
| --- | --- | --- |
| Antibodies | | |
| human CD4 (RPA-T4) eFlour 450 1:100 | eBioscience | 48-0049-41 |
| human CD8 (RPA-T8) PE-Cy7 1:400 | BD Pharmigen | 557750 |
| human CD69 (FN50) PE 1:100 | Biolegend | 310905 |
| human CD3 (OKT3) Brilliant Violet 510 1:100 | BD Pharmigen | 566779 |
| human CD45RA PerCP 1:100 | eBioscience | 45-0458-42 |
| mouse CD4 (RM4-5) eFluor 450 1:300 | eBioscience | 48-0042-80 |
| mouse CD8alpha (53-6.7) PE-Cy7 1:500 | eBioscience | 25-0081-81 |
| mouse CD69 (H1.2F3) PerCP-Cy5.5 1:200 | eBioscience | 45-0691-80 |
| mouse CD3 (17A2) Brilliant Violet 510 1:300 | Biolegend | 100233 |
| mouse CD24 (M1/69) PE 1:800 | eBioscience | 12-0242-81 |
| mouse TCRß (H57-597) PE 1:800 | eBioscience | 12-5961-81 |
| mouse CD25 (PC61.5) PE-Cy5 1:400 | eBioscience | 35-0251-80 |
| mouse CD44 (IM7) eFlour 506 1:100 | eBioscience | 69-0441-80 |
| mouse CD5 (53-7.3) PE-Cy5 1:200 | Biolegend | 100609 |
| mouse CD127 (A7R34 | eBioscience | 11-1271-81 |
| mouse TCRgd (eBioGL3) APC 1:400 | eBioscience | 17-5711-81 |
| mouse CD27 (LG.7F9) SuperBright 436 1:200 | eBioscience | 62-0271-80 |
| mouse FoxP3 (FJK-16S) APC 1:200 | eBioscience | 17-5773-80 |
| mouse CD62L (MEL-14) APC 1:200 | eBioscience | A14720 |
| mouse CD25 (PC61.5) PE 1:400 | eBioscience | 12-0251-81 |
| mouse CD19 (eBio1D3) APC 1:500 | eBioscience | 17-0193-80 |
| Mouse Ki67 (16A8) AlexaFluor 647 1:200 | Biolegend | 652407 |
| Anti-Mouse TCR Vß Screening Panel | BD Biosciences | 557004 |
| *Phaseolus vulgaris* Leucoagglutinin FITC 1:1000 | Vector Laboratories | FL-1111-2 |
| *Galanthus nivalis* lectin FITC | Vector Laboratories | FL-1241-2 |
| *Sambucus nigra* lectin Cy5 1:1000 | Vector Laboratories | CL-1305-1 |
| *Lycopersicon esculentum* lectin DyLight594 1:1000 | Vector Laboratories | DL-1177-1 |
