# Supplementary figures and images for: Mannosylated glycans impair normal T-cell development by reprogramming commitment and repertoire diversity
Source: Cell Mol Immunol. 2023 Jun 21;20(8):955–68. doi: 10.1038/s41423-023-01052-7 (PMC10387478; doi:10.1038/s41423-023-01052-7)

SuppFigure 1

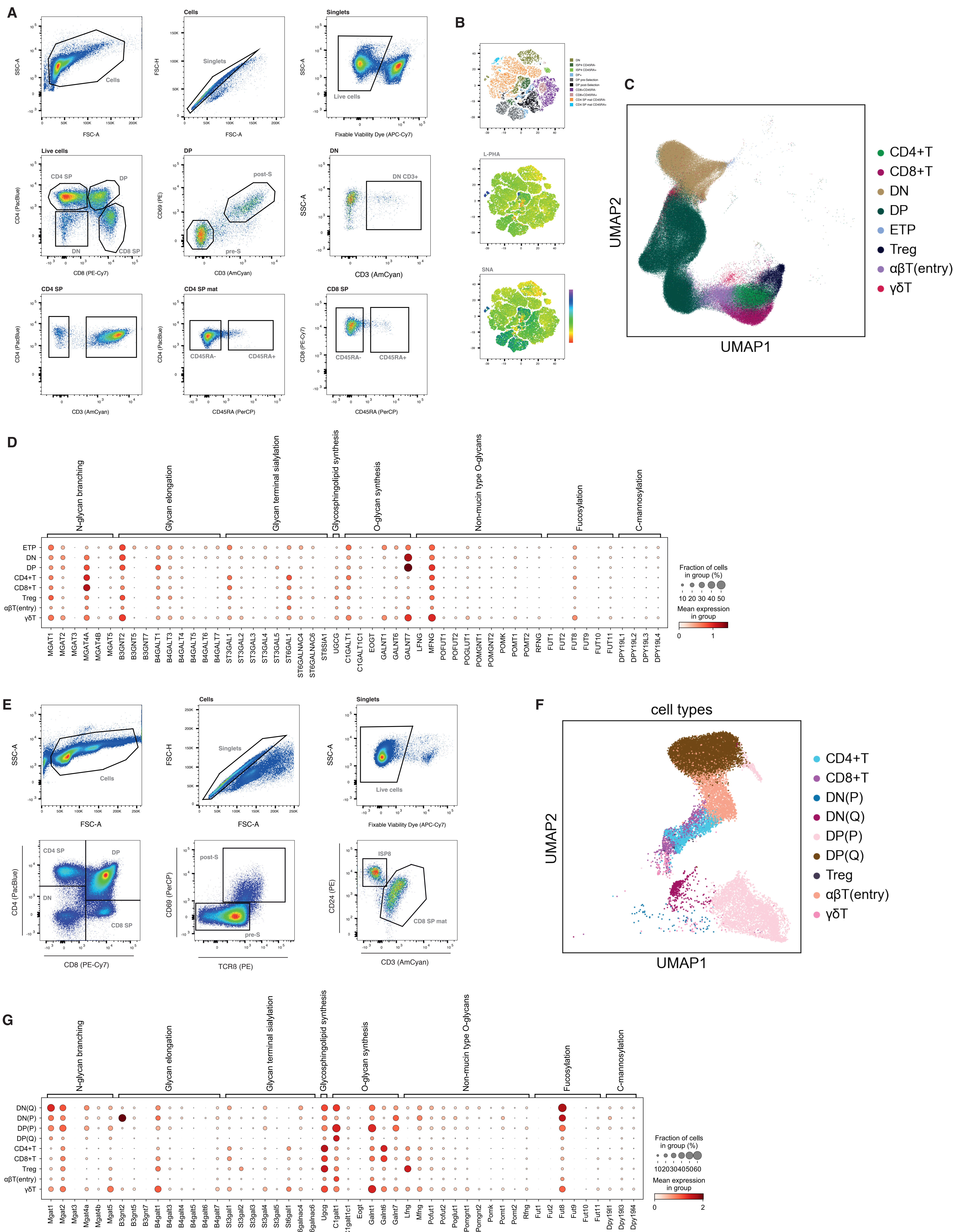

Supplement: Supplementary file 2 — Supplemental Figure 1 [file 41423_2023_1052_MOESM2_ESM.pdf]

SuppFigure 2

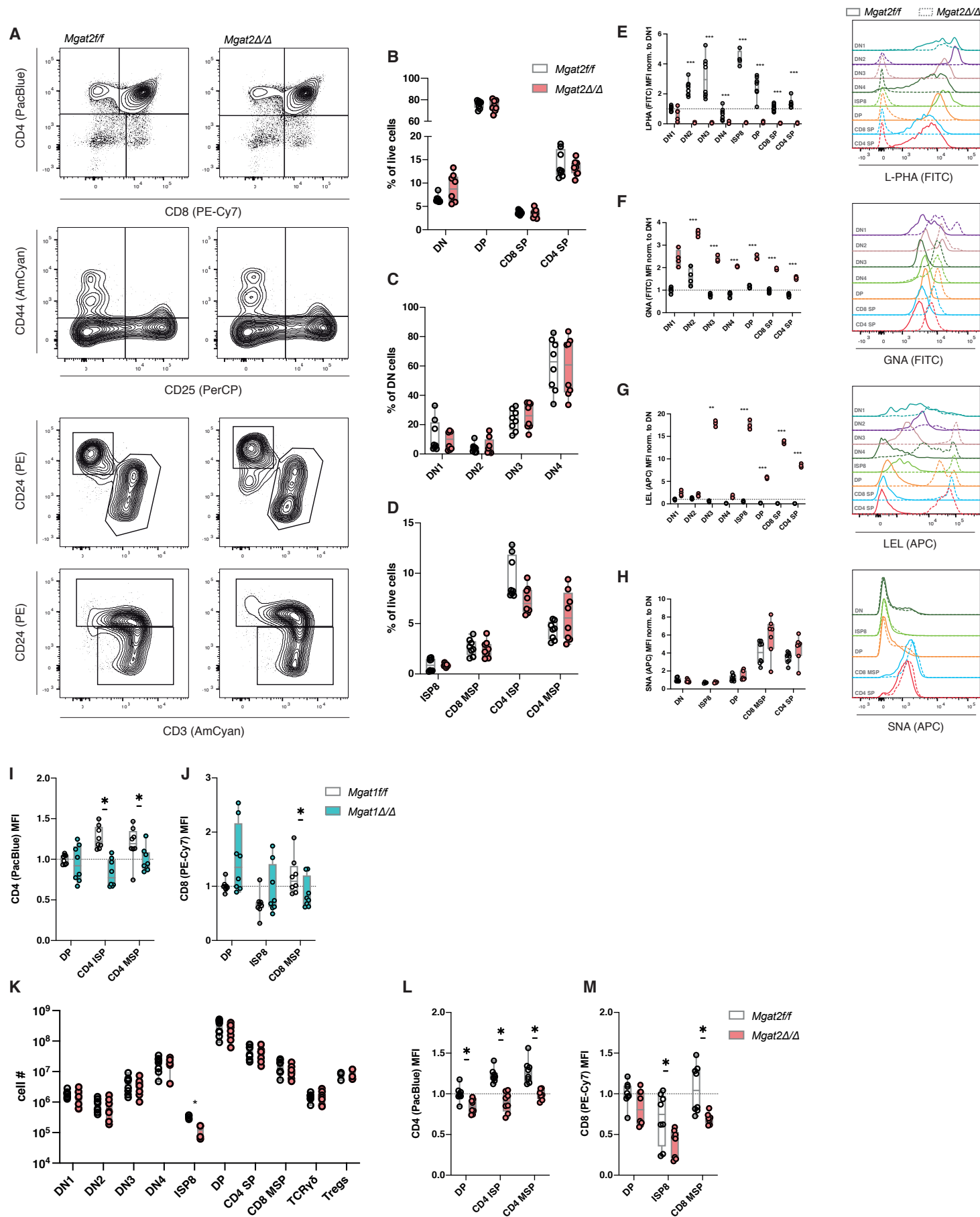

Supplement: Supplementary file 3 — Supplemental Figure 2 [file 41423_2023_1052_MOESM3_ESM.pdf]

SuppFigure 3

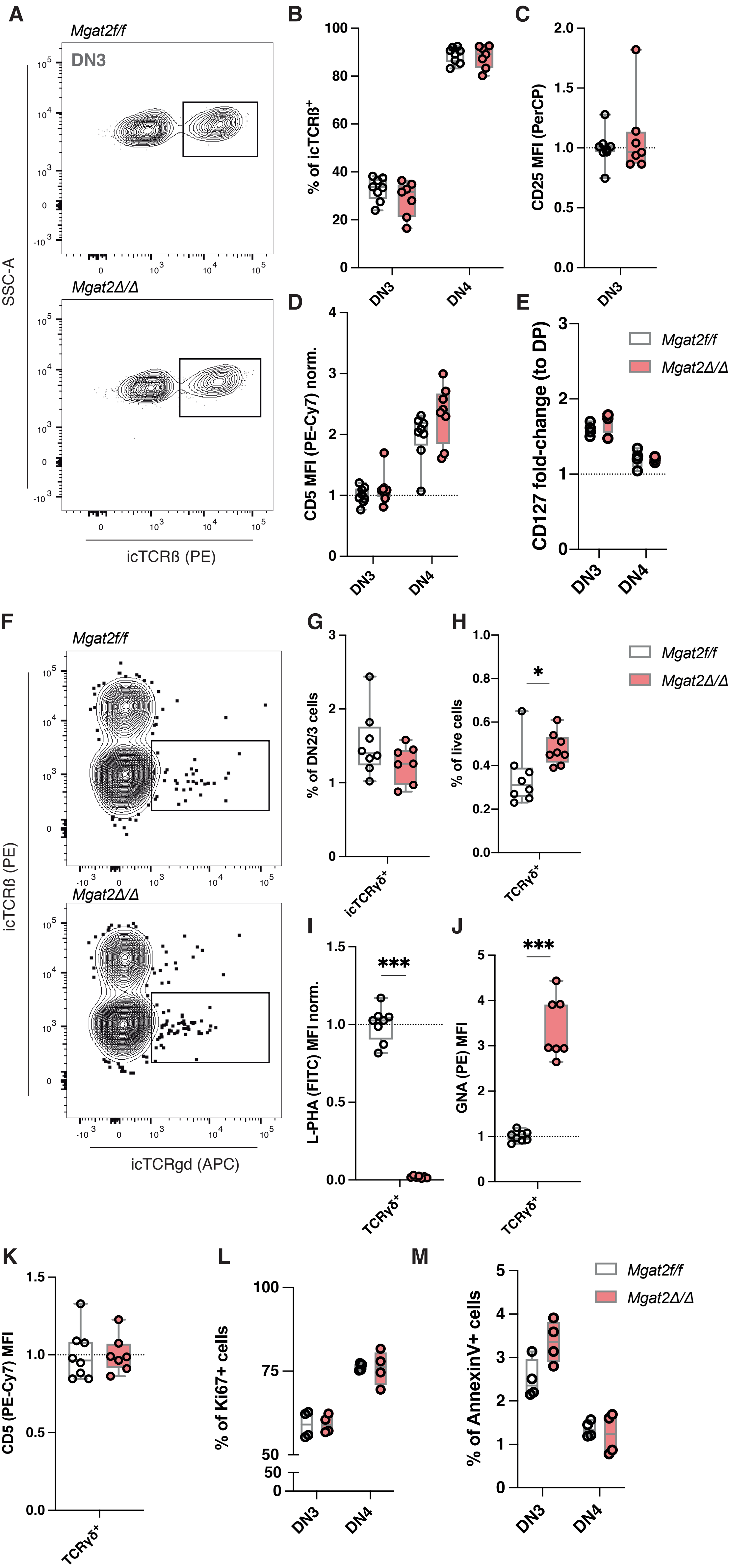

Supplement: Supplementary file 4 — Supplemental Figure 3 [file 41423_2023_1052_MOESM4_ESM.pdf]

SuppFigure 4

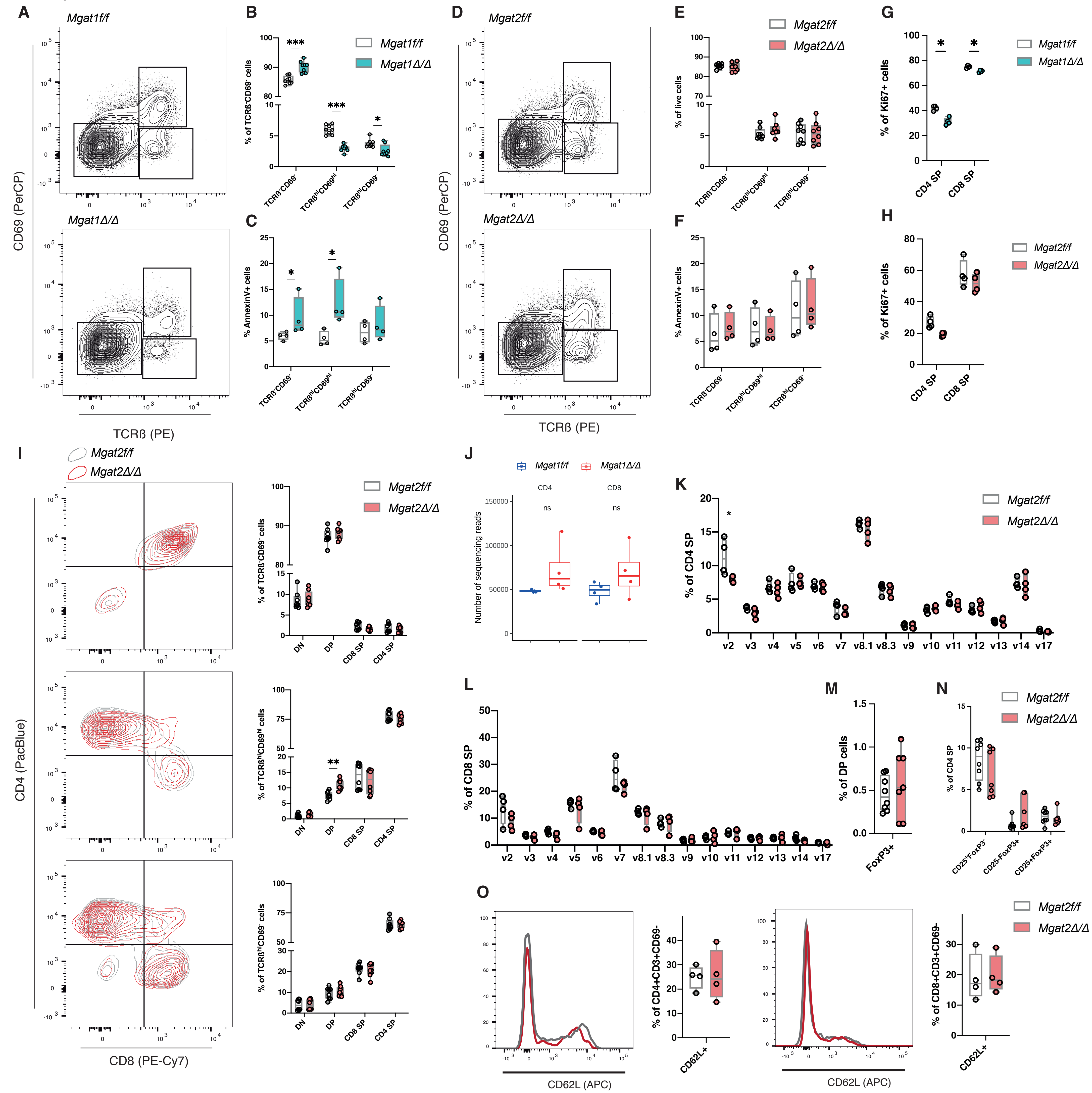

Supplement: Supplementary file 5 — Supplemental Figure 4 [file 41423_2023_1052_MOESM5_ESM.pdf]

SuppFigure 5

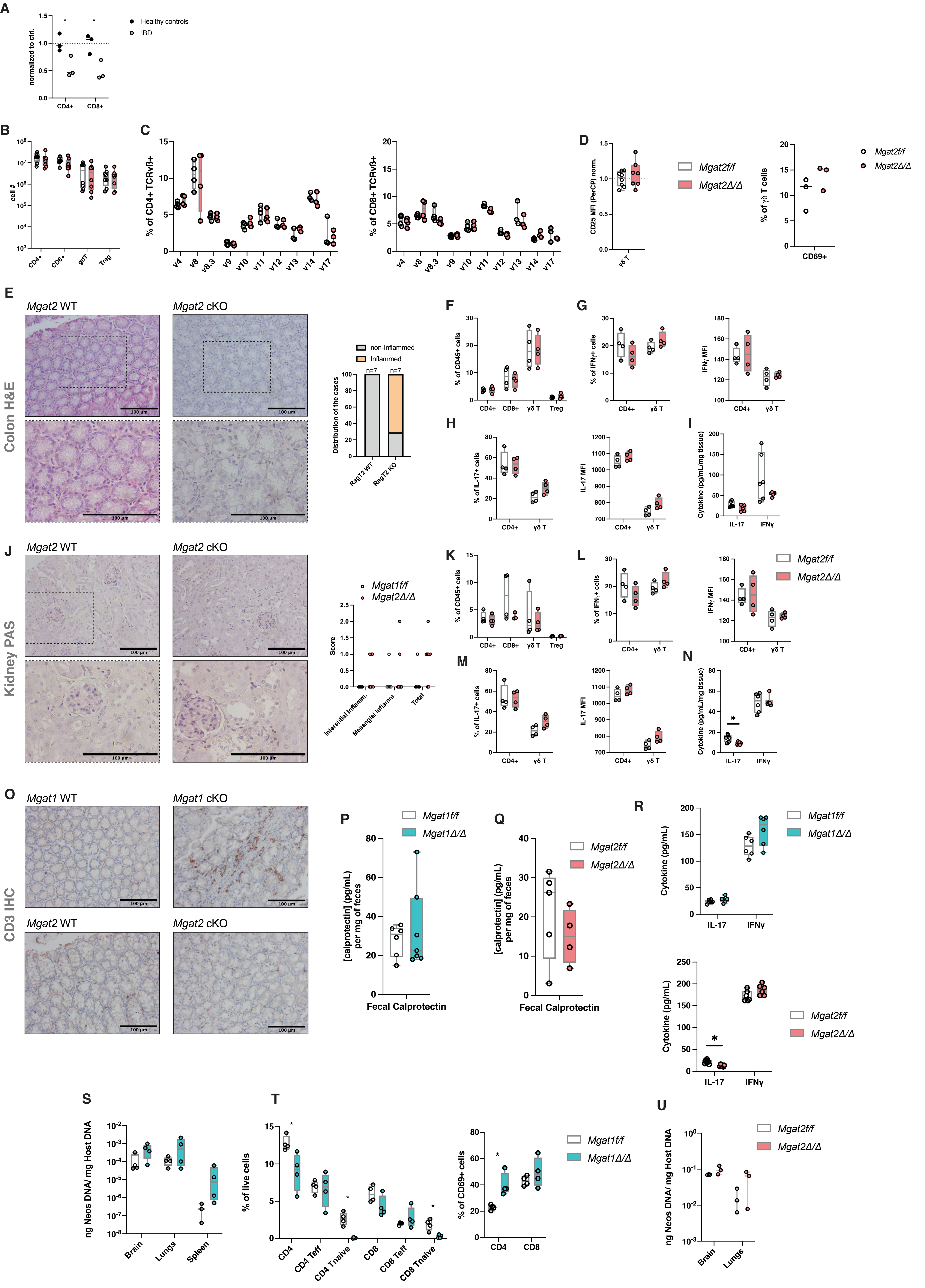

Supplement: Supplementary file 6 — Supplemental Figure 5 [file 41423_2023_1052_MOESM6_ESM.pdf]
